# Factors influencing seasonal chemistry patterns in Virginia mountain streams

**DOI:** 10.1007/s10533-024-01163-x

**Published:** 2024-08-06

**Authors:** Ami L. Riscassi, Todd M. Scanlon, James N. Galloway

**Affiliations:** Environmental Sciences Department, University of Virginia, Charlottesville, VA USA

**Keywords:** Seasonality, Hydro-biogeochemistry, Streams, Forested watershed, Acidification

## Abstract

The relative influence of seasonal patterns in hydrological flow and seasonal differences in biological and geochemical activity on stream chemistry patterns is difficult to discern because they covary; temperate systems are characterized by lower mean flow in the summer (i.e. corresponding to deeper flow paths, elevated temperature, and biological activity), and higher mean flow in the winter (i.e. corresponding to shallower flow paths, depressed temperature, and biological dormancy). Using 2018 data, when seasonal stream flow conditions reversed, and two prior conventional water years, the relationship between monthly acid-relevant analyte concentrations and streamflow were compared within and between winter and summer to provide insight into controls on characteristic seasonal chemistry patterns at two mid-Appalachian sites with distinct geology (weatherable mafic and weather resistant siliciclastic). Acid neutralizing capacity (ANC) increased (1) with lower flow, in both seasons and (2) in summer, for all flow conditions. The compounding impacts resulted in a doubling of concentration from typical winter with high flow to summer with low flow at both sites. Base cation patterns tracked ANC at the mafic site, resulting in an ~ 60% increase of from winter with high flow to summer with low flow; distinctions between summer and winter contributed more to the seasonal pattern (72%) than changes in flow. Sulfate increased at the mafic site (1) with higher flow, in both seasons and (2) in winter, for all flow conditions, resulting in an ~ 50% increase from summer with low flow to winter with high flow; distinctions between winter and summer conditions and flow contributed similarly (40–60%) to the typical seasonal chemical pattern. The biogeochemical mechanism driving differences in stream chemistry between summer and winter for the same flow conditions is likely increased rates of natural acidification from elevated soil respiration in summer, resulting in greater bedrock weathering and sulfate adsorption. Findings highlight the significance and consistency of growing vs dormant season variations in temperature and biological activity in driving intra-annual patterns of stream solutes. This data set informs parameterization of hydro-biogeochemical models of stream chemistry in a changing climate at a biologically relevant, seasonal, timescale.

**Supplementary Information:**

The online version contains supplementary material available at 10.1007/s10533-024-01163-x.

## Introduction

A defining characteristic of temperate deciduous forests is distinct seasonality (Adams et al. 2019; Fusco et al. 2024). Driven by the annual cycles of photoperiod and temperature, forest vegetation goes through well-defined and approximately equal length warm ‘growing’ and cold ‘dormant’ periods (Korner et al. 2023). The annual cycle of deciduous forest evapotranspiration, paired with precipitation throughout the year (excluding humid subtropical climates) drives a distinct seasonal streamflow pattern, with lower mean flow in the summer, growing season and higher mean flow in the winter, dormant season (Ohte and Tokuchi 2011). Chemical elements from soil, bedrock, forest vegetation, and atmospheric deposition, are transformed within the terrestrial environment through physical, chemical, and biological processes. These processes are influenced by seasonal cycles of temperature, light, and the presence and amount of water (Cronan 2018; Levia et al. 2011; Likens 2013). Stream water chemistry may also have a characteristic seasonal pattern reflecting both the changing availability of solutes due to differing rates of biogeochemical activity and/or the hydrologic transport conditions that variously connect the deeper regolith or shallow soils to the stream with shifting flow paths (Stewart et al. 2021). In turn, the life cycles of aquatic biota respond and are adapted to these predictable seasonal fluctuations (i.e., aquatic phenology) being more or less susceptible to disturbance or stress, such as extreme temperature, acidic conditions and floods, at various life stages (Blum et al. 2018). Despite the seasonal baseline underlying many aspects of a temperate watershed, there is a notable deficit in our understanding of the relative contributions of seasonal differences in hydrological flow as compared to seasonal variations in biological activity and temperature in shaping seasonal stream water chemistry patterns.

The chemical stream water response to, and subsequent recovery from, acid deposition has been a focus of water quality monitoring efforts in the Eastern U.S. for the past five decades (Burns et al. 2006; Mast 2013; Fuss et al. 2015; Kline et al. 2016; McHale et al. 2017; Scanlon et al. 2021). Stream acidity exerts a dominant control on key ecological variables such as fish species richness, density, biomass, and brook trout abundance (Jastram et al. 2013; Baldigo et al. 2019; Harmon et al. 2021). Stream water sampling in support of biological assessments in acid impacted streams is typically focused on the season with higher mean flow conditions, considered to be the most acidic due to shifting flow paths that dilute base cations while mobilizing acid anions (Baldigo et al. 2019). In temperate regions, the higher flow season (i.e. average conditions characterized by monthly flow) corresponds to non-growing periods when snowmelt dominates and/or evapotranspiration has been diminished for an extended period and conversely, the summer growing season is typified by persistent low flow conditions due to maximal evapotranspiration (Swank and Waide 1988; Baily et al. 2003; Adams et al. 2012; Lutz et al. 2012; Aulenbach and Peters 2018). Storm events, which generate peak flows and associated changes in stream chemistry (e.g. episodic acidification) over a period of hours, occur throughout the year and are distinct from the seasonal baseline pattern of streamflow and chemistry (Wigington et al. 1990). Recent assessments of long-term trends in stream chemistry in the Appalachian region have documented a reduced severity in acid episodes in contrast to the absence of change observed during more moderate and lower flow conditions (Riscassi et al. 2019; Scanlon et al. 2021). The lack of improvement in chronic conditions highlights the need to gain more insight into controls on underlying seasonal patterns.

**Fig. 1:**
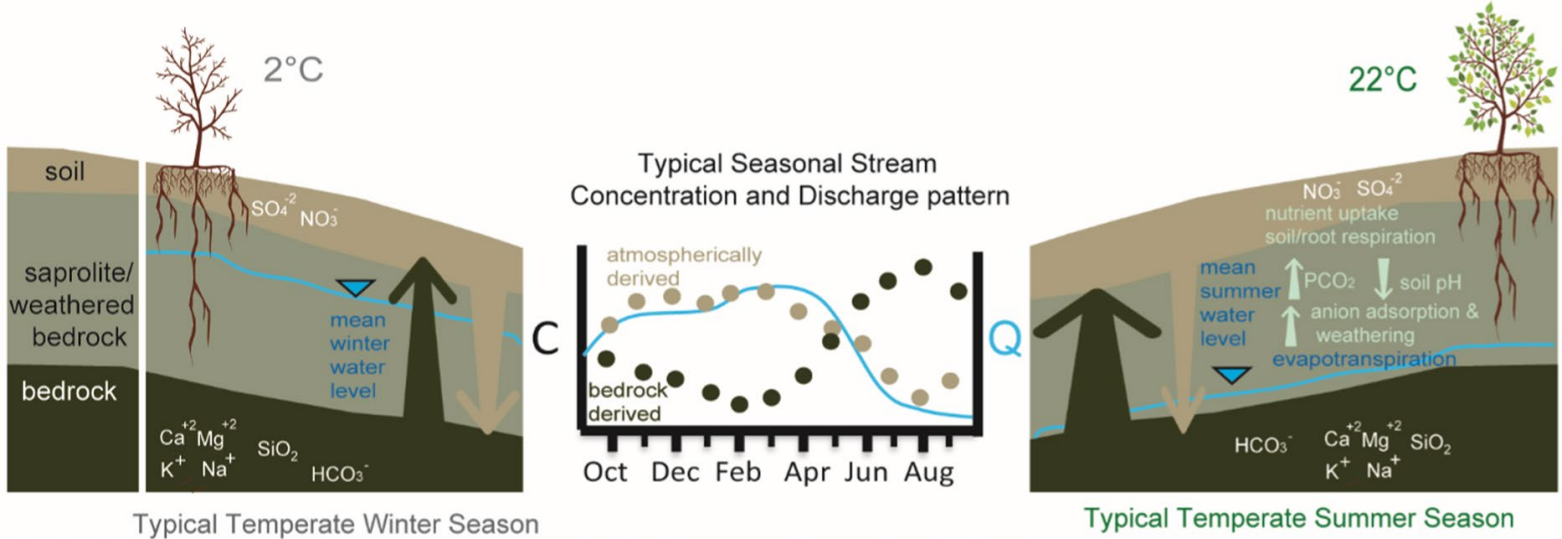
Conceptual subsurface cross section illustrating atmospherically derived solutes, sulfate (SO_4_^−2^) and nitrate (NO_3_^−^), and bedrock derived solutes, calcium (Ca^+2^), magnesium (Mg^+2^) potassium (K), sodium (Na), silica (SiO_2_), and bicarbonate (HCO_3_^−^). The arrow direction points away from the source. Arrow thickness indicates the relative abundance and availability of solutes for stream transport originating from surficial (light brown) or geologic (dark brown) sources. Changes in relative abundance of analytes can occur with depth, due to vertical variations in sources, as well as between seasons, due to variations in biological activity. In comparison to winter (left panel), summer (right panel) conditions are associated with 1) lower streamflow, deeper flow paths enriched in bedrock derived solutes, and 2) nutrient uptake and soil and root respiration resulting in decreased soil pH. Morea acidic soil conditions can reduce availability of anions via increased adsorption and increase availability of bedrock derived solutes via increased weathering. The center panel illustrates the corresponding seasonal pattern for monthly stream discharge (Q, blue line) and monthly flow-weighted solute concentration for surficial (C, light brown) and geologic (C, dark brown) sources

The elevated concentration of bedrock-derived solutes in summer has been attributed to seasonal hydrological processes as lower flows connect the stream to areas below the regolith with greater amounts of those solutes (Rice and Bricker 1995) or by the influence of flow paths on weathering rates (Horton et al. 1999; Douglas 2006; Li et al. 2020). The depressed concentrations of sulfate, the primary acidifying agent in watersheds affected by acid deposition, observed in summer (Driscoll et al. 1989; Lynch and Corbett 1989; Shanley and Peters 1993; Huntington et al. 1994; Rice and Bricker 1995; Peters et al. 1999) has been attributed to lower flow conditions which mobilize analytes closer to bedrock, with chemical signatures distinct from those in the upper soil horizons. Seasonal chemical patterns for acid-relevant analytes are frequently attributed to variation in streamflow as well as the biogeochemical reactions associated with changes in those flow conditions. For example, increased stream sulfate concentrations in fall have been attributed to soil sulfate oxidation during summer drought followed by transport to the stream during fall storm events (Mayer et al. 2010). The role of seasonal differences in biogeochemical processes independent of hydrology have also been noted. Through an experimental ecosystem (i.e., Hubbard Brook Experimental Forest Sandbox Experiment), Berner et al. (1998) found strong evidence that temperature dependent weathering is a dominant cause of increases in summer cation concentrations. Also evaluating data at Hubbard Brook, Nodvin et al. (1988) suggested that depressed summer sulfate concentrations may be influenced by increasing adsorption rates from soil acidification in the growing season. In contrast to sulfate, evaluations of stream nitrate patterns consistently consider seasonal differences in biological processes. Nitrate is both an essential plant nutrient and influenced by hydrology in both transport and biogeochemical reactions as summarized for 25 temperate forests in North America, Europe, and Japan (Ohte et al. 2010). A simple schematic of the characteristic subsurface water level, temperature, and differences in biological and geochemical activity for summer and winter in temperate climates is provided in Fig. 1.

**Fig. 2:**
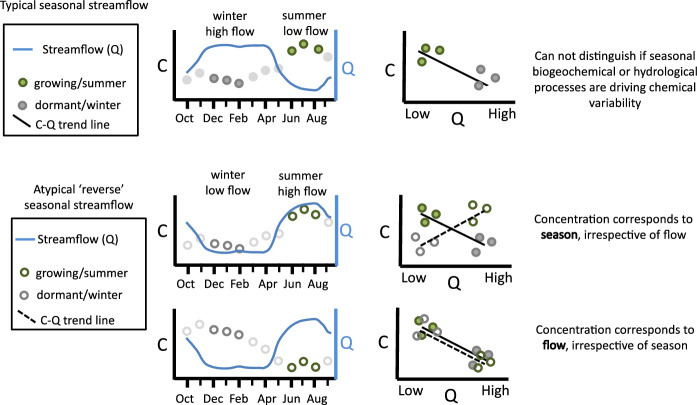
Conceptual diagram of monthly stream discharge (Q) and monthly flow-weighted stream chemical concentration (C) over time and corresponding plot of Q vs C for a bedrock derived solute. The upper panel demonstrates the challenge of identifying drivers of seasonal-concentration variations in a typical temperate climate (summer with low flow and winter with high flow). The lower two panels illustrate how incorporating analysis of stream chemistry during an atypical year when seasonal streamflow is reversed (summer with high flow and winter with low flow) can provide information to decipher drivers, with end member examples of seasonal biogeochemical processes dominating (middle panel) and hydrological flow dominating (lower panel) presented

Distinguishing the relative influence of seasonal differences in biogeochemical processes and hydrological flow on stream chemistry is difficult at the watershed scale because the cycles of each are interconnected via climate and plant physiology. This challenge is illustrated through an example concentration-discharge (C–Q) plot (Fig. 2, upper panel), derived from monthly streamflow (Q) and bedrock-derived analyte concentration (C), frequently used to assess hydrological controls on stream chemistry, though typically at hourly or daily resolution (Stewart et al. 2021). In this example, data are additionally distinguished by season illustrating elevated concentrations during mean monthly low flow conditions, co-occur with the summer season. One way to identify the relative influence of growing and dormant season differences in biogeochemical activity as compared to hydrological flow is to observe concentrations within each season for the complete range of monthly flow conditions typically observed over an annual cycle. If stream concentrations remain consistent within a season, regardless of flow condition, then seasonal biogeochemical drivers prevail (Fig. 2, middle panel). In contrast, if concentrations track with streamflow representing subsurface flow-paths and associated vertical variation in solutes, irrespective of season, then hydrological flow and associated source area controls dominate (Fig. 2, lower panel). Fortuitously, in the 2018 water year (defined as the 12-month period starting from Oct. 1 of preceding year through Sept. 30), consistent drought conditions throughout the dormant season were followed by frequent precipitation during the growing season, mimicking a monsoon-like rainfall pattern. The resulting reversal in the typical seasonal monthly stream flow pattern in regularly monitored headwater streams allowed for investigation of the influence of seasonal changes in biogeochemistry alongside hydrology on stream chemistry patterns. Furthermore, insights into likely seasonal biogeochemical controls in these same watersheds can be drawn from prior observations of stream chemical response to ecological disturbance (Webb et al. 1995; Eshelman et al. 1998).

In this study we use a unique data set to gain insight into controls on seasonal stream composition in two mid-Appalachian forested mountain watersheds representing end members with respect to acidification sensitivity. The primary objectives of this research are to (1) quantify the relative role of variations in seasonal biogeochemical processes and hydrological flow on characteristic seasonal chemical patterns, (2) gain insight into the likely biogeochemical mechanisms driving those patterns, and (3) determine the influence of bedrock composition on drivers of seasonal stream chemistry. In addressing these objectives, we seek to improve the fundamental understanding of factors that contribute to intra-annual patterns of individual analytes including sulfate, nitrate, chloride, base cations, silica, and acid neutralizing capacity. Evaluating the influence of changes in monthly streamflow within a season, will allow for more accurate forecasting of the stream chemical response to a future climate, as models predict alterations in precipitation, and corresponding streamflow, will vary on a seasonal timeframe (IPCC, 2014, 2022; Moustakis et al. 2021).

## Site descriptions

**Fig. 3:**
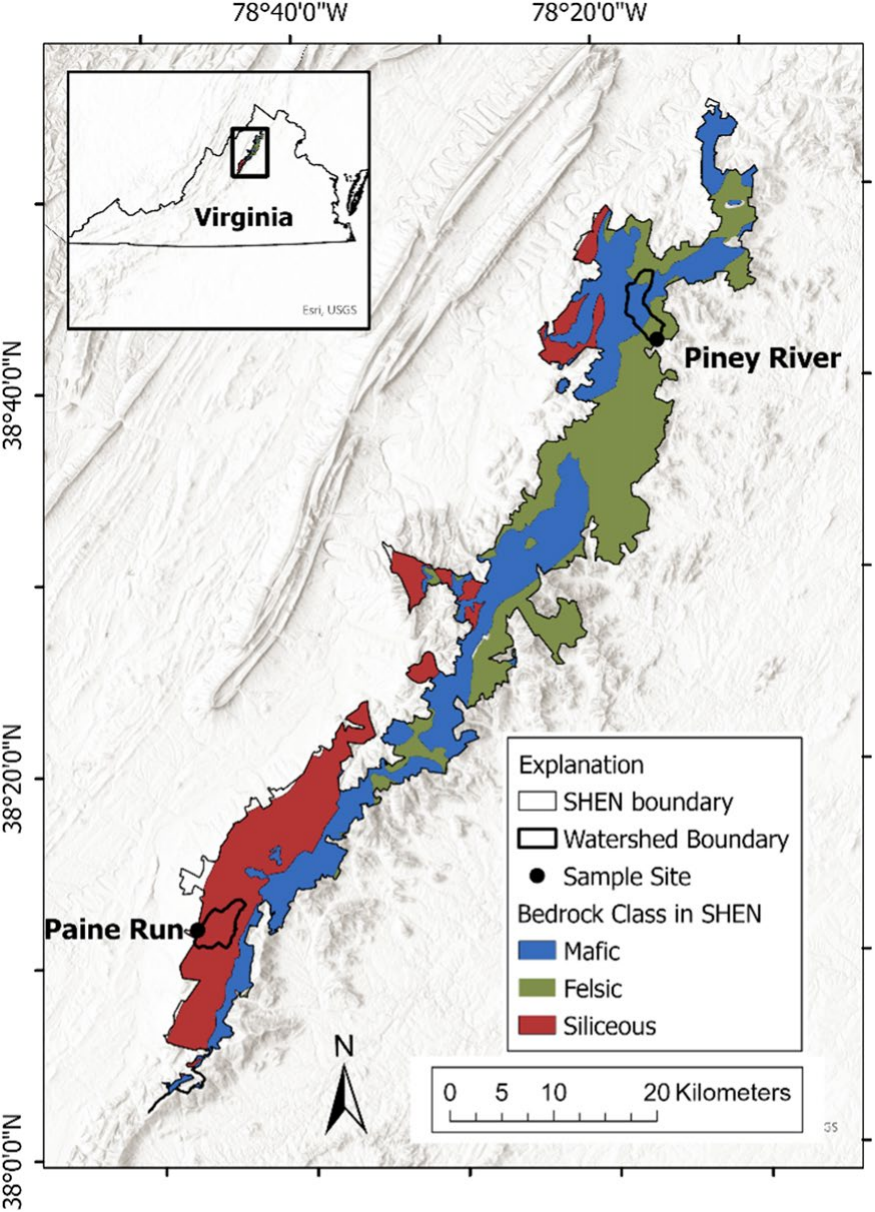
Map of study watersheds and sampling sites, distinguished by bedrock composition, within Shenandoah National Park (SHEN) in western Virginia

The two study watersheds, Piney River and Paine Run, are located within the north and south management districts, respectively, of Shenandoah National Park (SHEN) which overlies the crest of the northern Blue Ridge Mountains in western Virginia (Fig. 3). Elevations within SHEN peak at ~ 1,200 m on the ridge and descend to ~ 150 m in the foothills. The lower elevations have a modified continental climate, and higher elevations have cooler temperatures overall; mean annual temperatures are 12 °C in lowlands compared to 9 °C at elevation (Sullivan et al. 2003). Precipitation is evenly distributed throughout the year with stream discharge lowest in August and September, following peak evapotranspiration. The baseflow is consistently elevated in December through March and brief storm flows can occur throughout the year.

Analytes in these streams include base cations as well as carbonate solutes (measured as acid neutralizing capacity, ANC) derived mainly from geologic weathering, and acid anions, mainly sulfate, derived from atmospheric deposition and subsequent soil retention and release. The acid–base chemistry of stream water in SHEN watersheds is closely tied to the soils and underlying bedrock geology. Shenandoah National Park is comprised of three major geologic types, which range from least sensitive to acid deposition (mafic) to most sensitive (siliciclastic; Gathright 1976). Sulfate is the dominant acid anion associated with acidic streams, both in SHEN and within the central Appalachian Mountain region (NAPAP 1991; Webb et al. 2004). Nitrogen is typically tightly cycled in these systems, with nitrate concentrations at or near analytical detection limits (< 0.2 µeq L^−1^), reflecting stage 0 of nitrogen saturation based on criteria established by Traaen and Stoddard (1995). Exceptions occur during transient disturbance such as large-scale defoliation events (Eshleman et al. 1998). During these disturbance periods stream nitrate concentrations increase, potentially reflecting stage 1 nitrogen saturation, with varying recovery rates tied to watershed hydrological processes (Riscassi and Scanlon 2009).

The study watersheds are headwater systems of similar size (11–12 km^2^), and each have land cover dominated by deciduous forest (Young et al. 2006; Table 1). Piney River and Pain Run watersheds are underlain by mafic and siliciclastic bedrock, respectively, and represent the endmembers in response to acid deposition in this region (Lynch and Dise 1985; Robison et al. 2013). In addition to hourly discharge, the two study sites have been monitored for water chemistry on a weekly basis and bi-hourly during storm flow since 1992, as part of the Shenandoah Watershed Study – Virginia Trout Stream Sensitivity Study (SWAS-VTSSS) long-term water quality monitoring and research program (Riscassi et al. 2021). Additional water chemistry data, collected once in each season (i.e., quarterly frequency) at additional headwater streams underlain by mafic (4 sites) and siliciclastic (34 sites) bedrock throughout the region (Fig. S1) within the SWAS-VTSSS program are evaluated to support site specific findings.

**Table 1:** Characteristics of intensively monitored study watersheds. SOC, soil organic carbon. C:N, carbon to nitrogen ratio

	Piney River	Paine Run
Watershed Area, ha	1240	1260
Dominant Bedrock	Mafic	Siliciclastic
Watershed aspect, degrees from North	164	− 80
Elevation mean (min/max), m	689 (363/1059)	648 (426/1029)
Watershed slope, degrees	13.6	21.7
Soil hydraulic conductivity, mm h^−1^	15.2–50.8^a^	50.8–508^b^
Soil series, description	Myersville, silt loam with a silty clay loam to clay subsoil^a^	Craigsville, deep, well-drained, fine sandy loam^b^
Mean watershed SOC, g m^−2c^	4499	2390
Soil C:N ratio^d^	20	28
Dominant Vegetation^e^	Central Appalachian Montane Oak—Hickory Forest (Basic Type)	Central Appalachian/ Northern Piedmont Chestnut Oak Forest

^a^U.S. Department of Agriculture (1961)

^b^U.S. Department of Agriculture (1979)

^c^Stoken et al. (2016)

^d^Welsch et al. (2001)

^e^Young et al. (2013)

## Methods

All methods associated with field data collection and laboratory analysis were established within the SWAS-VTSSS program as detailed within the Quality Assurance Project Plan (SWAS-VTSSS, 2020). In this assessment, the winter season is represented by the months of December, January, and February and the summer season is represented by the months of June, July, and August. These definitions of winter and summer align with the National Atmospheric Deposition Program (NADP, 2022) and the periods of minimum and maximum air temperatures for the watersheds, respectively. All calculations were performed using either R Statistical software (version 4.3.3, R Core Team 2021) or MATLAB software (version 9.10.0.1684407, The MathWorks Inc., Natick, MA). Statistical significance was determined at* p* < 0.05.

### Sample collection and analysis

Stream water samples were collected weekly by manual ‘grab’ sampling and bi-hourly by stage actuated automated samplers (Teledyne ISCO® 2900, Lincoln, Nebraska, USA) when flows reached 5% exceedance for the respective season. A subset of the collected automated event samples, which included all samples on the rising hydrograph limb and 20% randomly selected from the falling hydrograph limb, were analyzed. Including 52 weekly samples, a total of 186, 109, and 198 samples were collected and analyzed for Paine Run and 169, 119, and 170 for Piney River for the 2016, 2017, and 2018 water years, respectively. All unfiltered samples were analyzed for acid neutralizing capacity (ANC), calcium (Ca^+2^), magnesium (Mg^+2^), potassium (K^+^), sodium (Na^+^), sulfate (SO_4_^−2^), nitrate (NO_3_^−^) chloride (Cl^−^), silica (SiO_2_), and conductivity. Descriptions of analytical procedures and sample handling are provided in Riscassi et al. (2019). Analytical detection limits for each analyte, except ANC, are calculated on an annual basis; all values were above analytical detection, except for nitrate (detection limits ranged from 0.11 to 0.20 µeq L^−1^ NO_3_^−^ for the study period). At Paine Run and Piney River, 24 and 26% of nitrate values were below detection, respectively.

Quality control (QC) was maintained in the field through duplicates and trip blanks. Analytical procedure QC was maintained through laboratory blanks, laboratory duplicates, and standard quality control checks throughout the analytical run. Sample data is validated by evaluations of the ion balance and the difference between measured and calculated specific conductivity. Overall laboratory quality assurance is determined by participation in inter-laboratory proficiency tests administered by Environment and Climate Change Canada (ECCC, IQM; 2023). Assessments of quality assurance are reported to Shenandoah National Park annually and uploaded to the Integrated Resource Management Applications (IRMA) portal (https://irma.nps.gov/DataStore/SavedSearch/Profile/2451). Discharge data is calculated from hourly stage data through a rating curve updated each year as described in Riscassi et al. (2021).

### Computations and statistical analysis

Stream analyte concentrations, as well as the relationships between concentration and discharge, in the two study watersheds measured since 1993 are significantly changing in time as the systems recover from acid deposition (Riscassi et al 2019; Scanlon et al. 2021). Therefore, to minimize differences in chemistry resulting from different stages of recovery, the two years immediately preceding the 2018 atypical water year were evaluated as the optimal candidates for comparisons. The cumulative precipitation and monthly specific discharge for the 2016–2017 water years were determined to be representative of the magnitude and seasonal pattern observed for the long-term average conditions. Hereafter, data for the 2016–2017 period will be referred to as representing the ‘typical’ seasonal hydrologic conditions.

The focus of this study is on seasonal, as opposed to short-term dynamics, so data are evaluated at monthly resolution for statistical trends and differences, subsequently described. While not used in the statistical evaluations, the relationship between instantaneous concentration (µeq L^−1^ or µmol L^−1^) and discharge (mm h^−1^) for both the growing and dormant season were also evaluated with the C–Q methods described below and presented in Supplemental Information (Figs. S4 and S5),

#### Monthly stream fluxes, flow-weighted concentrations, and C–Q dynamics

Stream solute fluxes were calculated using the “composite method” loading model available within the USGS- R/loadflex package (Appling et al. 2015). Briefly, the model uses a two-step approach which combines predictions from a regression model (LOADEST) with an empirical “residuals correction” or period-weighted approach. The composite method was selected because it can be run at hourly resolution and makes use of chemistry data from both weekly and high-frequency storm sampling. Hourly stream chemical concentrations were predicted by LOADEST from hourly discharge and regressions derived from weekly samples for each site and period (2016/2017 and 2018). In the second step of the model, the concentration estimates are corrected to match the observations, which include both weekly and high-frequency storm data. Hourly fluxes are computed with measured discharge and predicted concentration and aggregated to monthly resolution within the model. Monthly flow-weighted concentrations were calculated by dividing the total monthly analyte flux (kg month^−1^) by the total monthly stream discharge flux (L month^−1^) and converting to appropriate units for the respective analyte.

The relationships between monthly flow-weighted concentrations for individual analytes and monthly stream discharge were determined for each season with the basic power law fit of C = *a*Q^*b*^, where C represents concentration, Q represents stream discharge and* a* and *b* are fitted parameters. The exponent *b* represents the slope of the concentration-discharge (C–Q) relationship on logarithmic axes (log C = *b* log Q + log *a*). Statistical comparisons, including slope, intercept, and population marginal mean (PMM), were evaluated between winter and summer trend lines. Differences between seasonal C–Q trendline intercepts and PMMs characterize the impact of seasonal biogeochemical processes on the availability of solutes for transport within the subsurface. The C–Q trendline slopes for individual seasons characterize the influence of hydrological controls, independent of seasonal differences in biogeochemical processes.

**Fig. 4:**
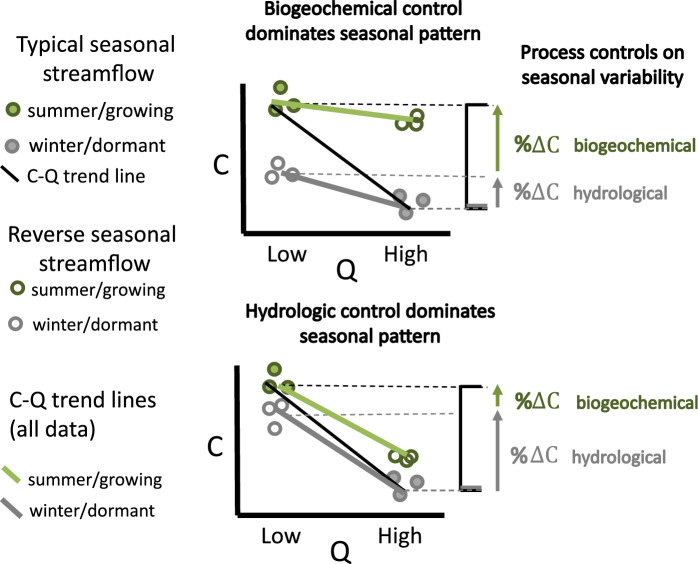
Diagram illustrating monthly streamflow (Q) and monthly flow-weighted concentrations (C) in the summer, growing season and winter, dormant season from a typical temperate (low flow in summer, high flow in winter) and atypical reverse pattern (low flow in winter and high flow in summer). The C–Q best fit line for the typical temperate year (black line) is shown for a bedrock-derived analyte (higher in summer with low flow, lower in winter with high flow). C–Q trend lines are determined for winter and summer. The contribution of changes in seasonal biogeochemical activity (% ΔC biogeochemical) and typical seasonal hydrologic variability (% ΔC hydrological) to typical seasonal chemistry patterns are quantified according to Eqs. (1) and (2). The top graph illustrates a system in which seasonal biogeochemical processes are more dominant in determining the seasonal pattern in a typical year, whereas the lower graph illustrates one in which hydrological flows are more dominant. Both axes are on a log scale

When both mean monthly streamflow and season were determined to have a significant independent impact on stream chemistry, their influences could be characterized as offsetting or compounding in a typical year. When compounding, a simple metric was used to attribute the percent of hydrological flow versus seasonal biogeochemical influence on the range of monthly flow-weighted concentrations. The annual concentration range during a typical year was defined as the difference between the concentration in summer during the lowest monthly flow and winter during the highest monthly flow of the evaluation period (denominator in Eq. 1). The range in concentration during winter from the lowest to highest monthly flow was considered to represent the fraction of total change attributable to hydrological flow (($\% \Delta C_{hydro}$), assuming the dormant season reflects minimal biogeochemical activity. The remaining concentration difference over a typical annual cycle is then attributed to seasonal variations in biogeochemical processes ($\% \Delta C_{biogeochem}$) as defined by equations listed below and illustrated in Fig. 4.

1 $$\% \Delta C_{hydro} = \frac{{\left| {C_{{{{winter, \,\,minlow}} }} {-} C_{{{winter, \,\,max \,\,flow}}} } \right|}}{{\left| {C_{{{summer, \,\,min \,\,flow }}} {-} C_{{{winter, \,\,max \,\,flow}}} } \right|}} \times 100$$

2 $$\% \Delta C_{biogeochem} = 100 - \% \Delta C_{hydro}$$

where, ${\text{C}}_{season, flow}$ = Monthly flow weighted concentration determined by the equation, C = *a*Q^*b*^, generated with data for the respective season (summer or winter), computed for minimum or maximum monthly flow (Q) observed during the study period. The coefficients (*a, b*) for each analyte and season are listed in Supplemental Table S1.

We acknowledge this simple metric is not comprehensive, as it does not quantify the role of hydrological flow during the summer or identify if the seasonal biogeochemical and hydrological flow influences on stream chemistry are offsetting. For example, if concentrations increase 50% from summer to winter and decrease 50% from low to high flow, in a typical summer with low flow to winter with high flow year, there would be no change in monthly concentration due to the offsetting impacts of streamflow and season. Despite these limitations, it is a useful metric to evaluate if one factor is dominant or if they are equivalent when influences are compounding. The attribution percentages are not intended to reflect certainty, but rather to be considered for their relative magnitudes as illustrated in Fig. 4

#### Annual fluxes and quarterly concentrations

Total atmospheric deposition of Ca^+2^, Mg^+2^, K^+^, Na^+^, Cl^−^, total N, and total S were computed and compared to annual stream fluxes for the three water years evaluated to illustrate intra-year variability and demonstrate which analytes were retained verses released from the watersheds. Detailed methods describing deposition calculations are reported in Supplemental Information (Text S1).

To determine if findings from the two intensive study sites are representative of the region, analyte concentrations for samples collected at 40 stream sites sampled quarterly were compared for the last week in January and the last week in July of the 2016/2017 and 2018 water years. Methods of data selection and analysis summarizing quarterly concentrations are reported in Supplemental Information (Text S2).

## Results

**Fig. 5:**
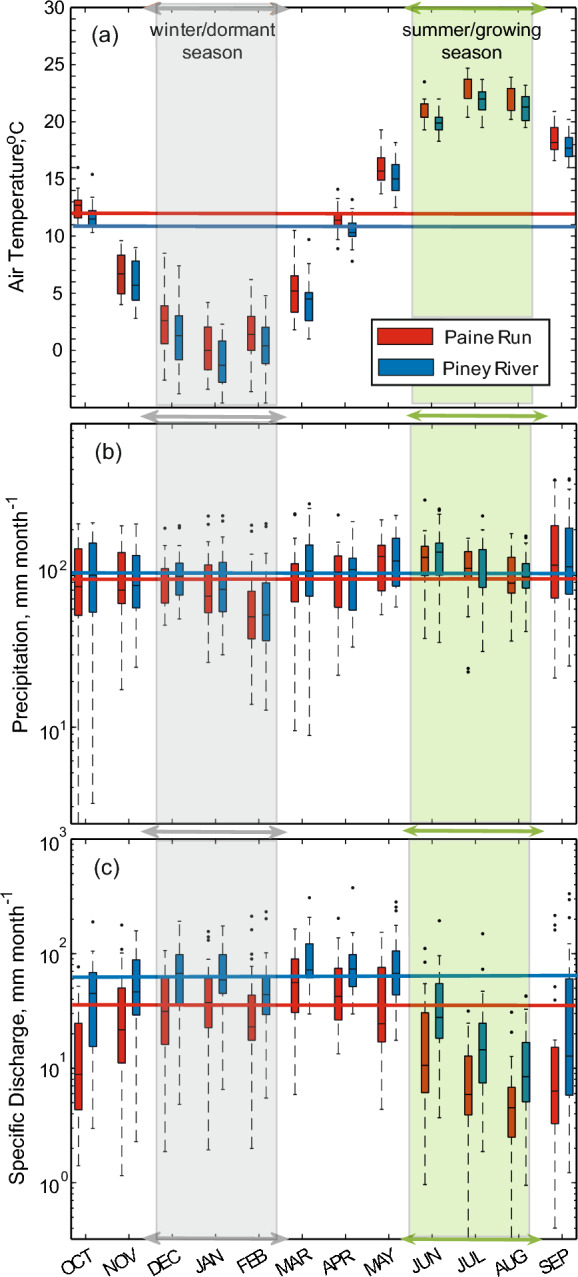
Box whisker plots (center line = median, box limits = 25 and 75th percentiles, outer lines = minimum and maximum, dots are outliers) of **a** monthly air temperature, **b** precipitation, and **c** specific discharge for the 1993–2017 water years at Piney River and Paine Run. Horizontal lines represent median annual values for the period, colored to match their respective site. The winter, dormant season (December, January, February) and summer, growing season (June, July, August), are indicated in each panel with grey and green arrows and shading, respectively. Monthly precipitation and air temperature data were obtained from the Parameter-elevation Regression on Independent Slopes Model (PRISM, http://prism.oregonstate.edu)

**Fig. 6:**
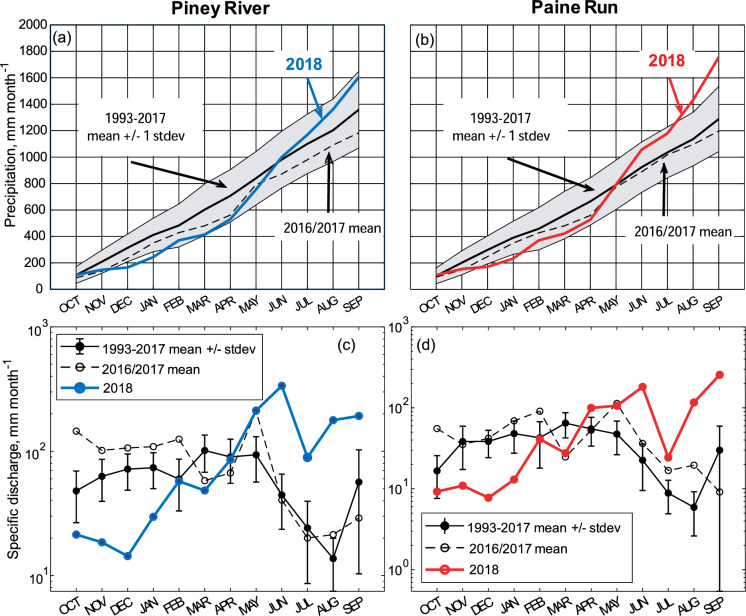
Cumulative mean monthly precipitation (± 1 standard deviation is represented by grey shading) for the 1993–2017 Water Year (WY) and for the 2016/2017 and 2018 WY for **a** Piney River and **b** Paine Run. Mean monthly discharge for the 1993–2017 WY and for the 2016/2017 and 2018 WY for **c** Piney River and **d** Paine Run. The 2016/2017 WYs are characterized as having a ‘typical’ climate while the 2018 WY is characterized by an ‘atypical’, monsoon-like precipitation pattern. Monthly precipitation and air temperature data were obtained from the Parameter-elevation Regression on Independent Slopes Model (PRISM, http://prism.oregonstate.edu)

Monthly air temperature, precipitation, and specific discharge at the two study sites for the 1993–2017 water years (Fig. 5) illustrate the characteristic pattern of maximum air temperature coincident with minimum monthly stream flow as a result of seasonal changes in evapotranspiration; precipitation has no seasonal pattern. Cumulative monthly precipitation graphs (Fig. 6a and 6b) illustrate the consistency in the interannual pattern (evident by the consistent slope of ~ 120 mm month^−1^) in the long-term record and the 2016/2017 years compared to the 2018 water year with a shallow slope (~ 67 mm month^−1^) in the fall/winter (October–March) period and a steep slope (~ 183 mm month^−1^) in the spring/summer (April–September) period. Monthly specific discharge at Piney River and Paine Run in 2016/2017 water years demonstrate the typical discharge pattern of high flow in the winter (~ 100 and 50 mm month^−1^, respectively) and relatively lower flows in the summer (~ 25 and 20 mm month^−1^, respectively). At both sites, 2018 represents a reversal from the long-term seasonal hydrologic pattern, with mean monthly summer streamflow similar to typical winter levels and winter mean monthly streamflow comparable to typical summer levels (Fig. 6c and d). Annual precipitation and stream fluxes are illustrated in Supplemental Information (Fig. S3).

**Fig. 7:**
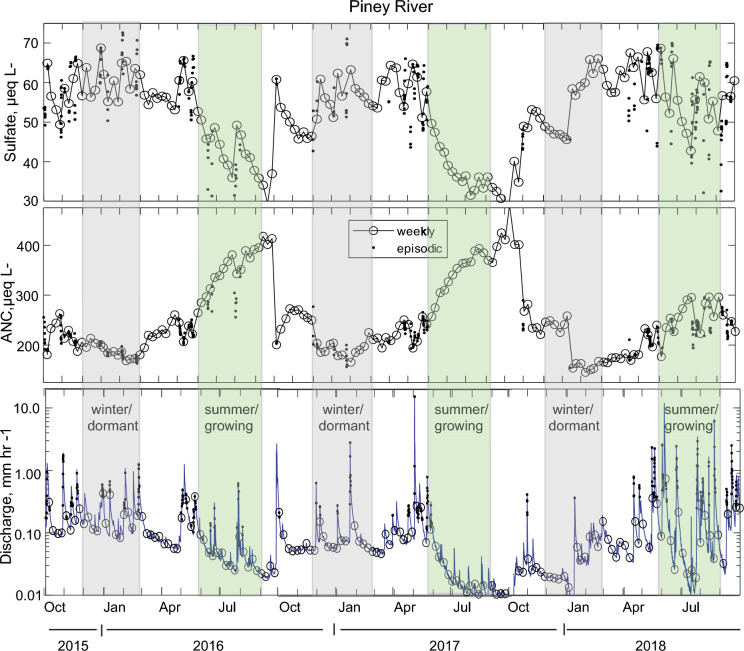
Sulfate concentration (upper panel), Acid Neutralizing Capacity concentration (ANC; middle panel) with weekly samples (open circles) and episodic samples (dots) and corresponding hourly stream discharge (lower panel) at Piney River for October 2015 through September 2018. The 2016–2017 water years were typical with respect to hydrology with higher flow in winter, dormant season (grey shading) and lower flow in the summer, growing season (green shading). The 2018 water year had anomalous ‘reverse’ seasonal hydrology. Note the magnitude of concentration change during brief high flow episodes, is less than the variability in the seasonal baseline

**Fig. 8:**
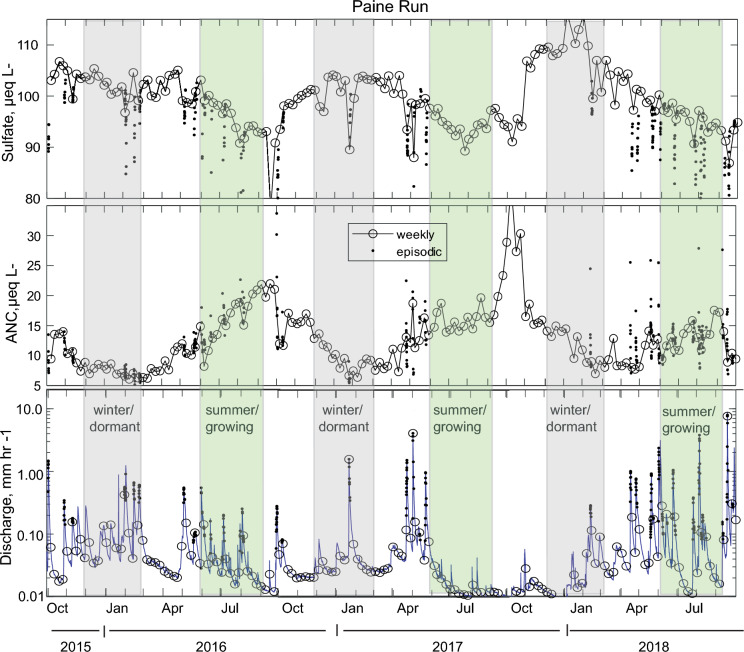
Sulfate concentration (upper panel), Acid Neutralizing Capacity concentration (ANC; middle panel) with weekly samples (open circles) and episodic samples (dots) and corresponding hourly stream discharge (lower panel) at Paine Run for October 2015 through September 2018. The 2016–2017 water years were typical with respect to hydrology with higher flow in the winter, dormant season (grey shading) and lower flow in the summer, growing season (green shading). The 2018 water year had anomalous ‘reverse’ seasonal hydrology. Note the magnitude of concentration change during brief high flow episodes, is less than or equal to the variability in the seasonal baseline

Times series of sulfate and ANC concentrations and stream discharge for the two study sites (Figs. 7 and 8) illustrate the seasonal patterns of characteristic atmospheric and bedrock derived solutes. In typical hydrologic years (2016–2017), there is a consistent pattern of elevated sulfate and depressed ANC in winter relative to summer. The atypical hydrologic year (2018) has less distinct seasonality overall, except for sulfate at Paine Run. The example time series demonstrates the need for an approach that summarizes and compares seasonal chemistry and hydrology to gain more quantitative insight.

**Fig. 9:**
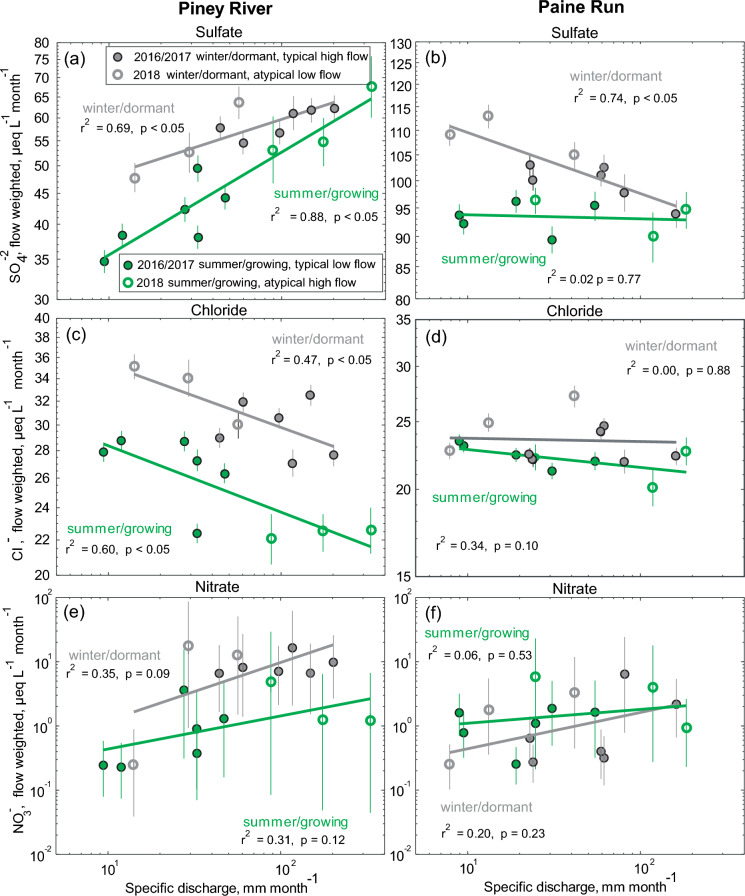
Monthly flow-weighted concentration versus monthly specific discharge for the summer, growing season and winter, dormant season for (a, b) sulfate, (c, d) chloride, and (e, f) nitrate at Piney River (left column) and Paine Run (right column). Error bars represent 95% confidence intervals. The best fit lines for the respective seasons, inclusive of both the typical (closed circles) and atypical (open circles) water years are presented along with the associated r^2^ and *p* values

**Fig. 10:**
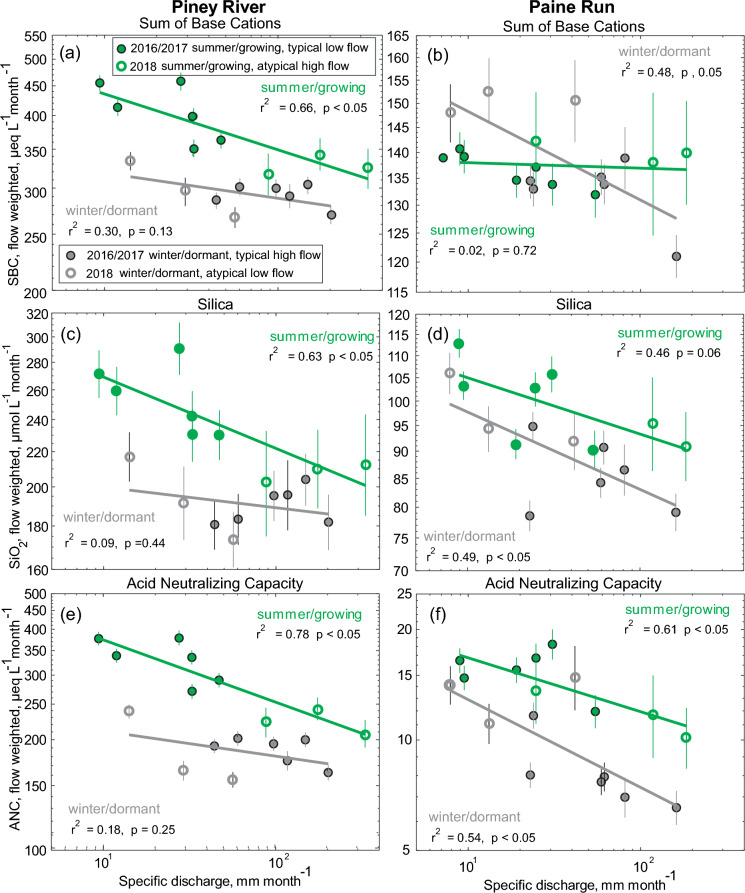
Monthly flow-weighted concentration versus monthly specific discharge for the summer, growing season and winter, dormant season for the (a, b) sum of base cations (SBC), (c, d) silica (SiO_2_), and (e, f) acid neutralizing capacity (ANC) at Piney River (left column) and Paine Run (right column). Error bars represent 95% confidence intervals. The best fit lines for the respective seasons, inclusive of both the typical (closed circles) and atypical (open circles) water years are presented along with the associated r^2^ and *p* values

Summer vs winter differences in monthly flow-weighted concentrations were characterized (consistently higher, lower, or inconsistent) and within season C–Q slopes were quantified (Table S1) and qualified (positive, negative, or chemostatic) for the same range of monthly hydrologic conditions typically observed over an annual cycle. Monthly flow-weighted concentrations of individual acid anions (sulfate, nitrate, and chloride) are typically lower in summer for the range of monthly flow conditions at both sites (Fig. 9), being statistically lower (p < 0.05) at mean flow (Table S1). Nitrate concentrations at Paine Run are the one exception to the pattern, with lower concentrations in winter (Fig. 9f, Table S1). Unlike the relatively consistent pattern between seasons (anions lower in summer), the C–Q relationships varied between analytes. For each season at Piney River, the C–Q relationship is positive (p < 0.05) for sulfate and nitrate (p > 0.05), while the relationship is negative (p < 0.05) for chloride (Fig. 9, Table S1). In contrast, at Paine Run, sulfate had a negative relationship with flow in winter (p < 0.05) and there was no significant relationship between nitrate or chloride and flow in either season, or for sulfate in summer (Fig. 9, Table S1). Monthly flow-weighted concentrations of base cations, silica, and ANC were typically higher in summer for the range of monthly flow conditions at both sites (Fig. 10), being statistically higher at mean flow (Table S1). The sum of base cation (SBC) concentrations at Paine Run are the one exception to the pattern, with lower summer concentrations at low flow and higher summer concentrations at high flow, resulting in no difference between seasons at mean flow (Fig. 10b, Table S1). At both sites, the direction of the C–Q relationships for SBC, silica, and ANC are negative for each season, though not all results are statistically significant (Fig. 10, Table S1). Patterns observed at the quarterly sites (Fig. S6) indicate trends observed at Piney River and Paine Run are representative of other mafic and siliciclastic sites in the region with one exception; the dilution pattern for sulfate in winter at Paine Run is not consistent with the pattern at other siliciclastic sites, which suggest no trend (Fig. S6e).

Typical seasonal variability, defined here as the change in mean monthly flow-weighted concentrations from the lowest flow in summer to the highest flow in winter was characterized for individual analytes. If the effect of flow and season on concentration were compounding (e.g. summer conditions and lower flow conditions both independently result in higher ANC concentrations) the relative amount of change was attributed to each as described in Fig. 4. For acid anions, typical seasonal patterns were distinct among analytes. Sulfate, the dominant acid anion, had a minimum concentration for summer with low flow, increasing to a maximum at winter with high flow at all mafic sites and at siliciclastic sites sampled quarterly; Paine Run is aseasonal and therefore considered an outlier. As both winter conditions and higher flow conditions independently result in elevated concentrations of sulfate, effects of seasonal biogeochemical processes and hydrological flow compounded to produce the typical seasonal pattern with changes in streamflow accounting for the majority (60%) of the change at Piney River (Table 2). Chloride did not have a distinct seasonal pattern at Piney River or Paine Run for the typical seasonal flow conditions. At Paine Run, the aseasonal pattern reflects the lack of change in concentration with flow or between seasons. In contrast, at Piney River the aseasonal pattern was due to equal and offsetting seasonal and hydrological dynamics. Chloride concentrations were ~ 20% higher in winter (Fig. 9c, Table S1), and ~ 20% lower for higher flow conditions (Fig. 9c). Seasonal nitrate patterns at Piney River were similar to sulfate, with minimum summer with low flow and maximum winter with high flow concentrations, with seasonal biogeochemical processes and hydrological flow compounding to produce the seasonal pattern. No seasonal dynamics were present for nitrate at Paine Run, though quarterly siliciclastic data illustrate higher summer with low flow, as compared to winter with high flow, concentrations (Fig S6e).

**Table 2:**
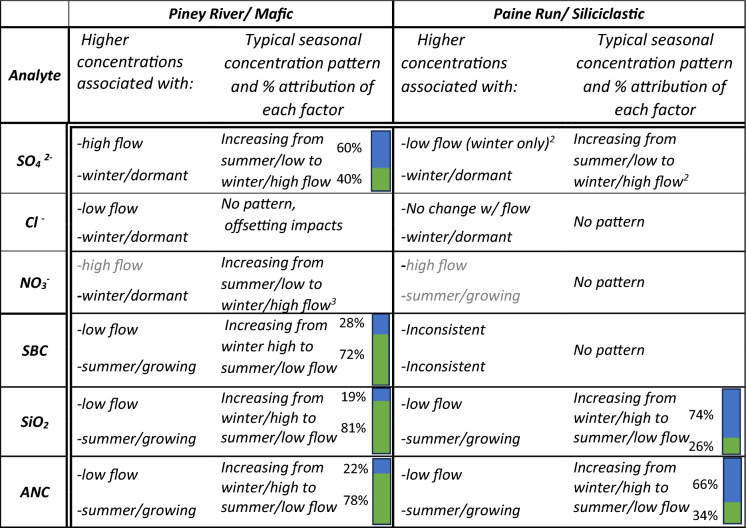
Summary of flow condition (low or high) and season (summer or winter) associated with higher analyte concentrations for the two study sites

Differences that are not statistically significant^1^ are presented in grey. The percent of both factors, hydrological flow (upper blue bar) and biogeochemical processes associated with seasons (lower green bar), attributed to the typical seasonal pattern when compounding, are shown to the left of the respective bar. Percentages were derived as described in Eqs. 1 and 2 and illustrated in Fig. 4. These values are intended to convey relative importance, not an exact attribution value

^1^Differences were considered significant 1) for flow if the C–Q slope for either season was statistically significant (Table S1) and 2) for season if the intercept for the C–Q lines or the concentration at mean flow were significantly different between seasons (Table S1)

^2^SO_4_^2−^ dynamics with flow at Paine Run (higher with lower flow) are not consistent with observations at quarterly siliciclastic sites indicating they may not be broadly applicable; therefore no attribution metric is calculated

^3^NO_3_^−^dynamics at Piney River are compounding, however the attribution metric was not appropriate as concentrations were near detection limit in summer

For bedrock derived solutes (silica, SBC, and ANC) typical seasonal patterns were generally similar with minimums during winter, and maximums during summer conditions; SBC at siliciclastic sites (both Paine Run and quarterly sites) is the exception with similar concentrations in both seasons. As both summer and low flow conditions independently result in elevated concentrations of these solutes, the role of seasonal biogeochemical processes and hydrological flow compound to produce the typical seasonal pattern (Table 2). Silica concentrations increased ~ 50% between winter with high flow and summer with low flow with 81 and 26% of the change attributed to differences in seasonal biogeochemical processes at Piney River and Paine Run, respectively. The SBC trends reflected silica trends with increases (60%) from winter with high flow to summer with low flow at Piney River. Seasonal biogeochemical processes (72%) compounded with hydrological flow to produce the seasonal SBC pattern at Piney River.

For the analytes evaluated, seasonal variability at both sites was greatest for ANC. Minimum bedrock derived solute and maximum acid anion concentrations, which both contribute to lower ANC, occur in typical winter with high flow conditions. Concentrations of ANC more than doubled from winter with high flow to summer with low flow at both Piney River (166 to 378 µeq L^−1^) and Paine Run (6 to 17 µeq L^−1^). Approximately 78 and 34% of the seasonal pattern in ANC is attributed to differences in seasonal biogeochemical activity at Piney River and Paine Run, respectively, with the remaining amount attributable to changes in hydrological flow (Table 2).

## Discussion

Field observations of streamflow and stream water chemistry were used to gain insight into how differences in seasonal biogeochemical activity and seasonal hydrological flow act in concert to produce characteristic stream chemistry patterns. Biogeochemical functioning is dependent on, and intricately linked to, hydrological dynamics via factors such as horizontal and vertical connectivity, water storage and release, water/rock contact time, generation of oxic and anoxic conditions, and transition zone locations in the subsurface (Li et al. 2021 and references therein). However, here we draw attention to biogeochemical activity that is necessarily distinct between winter and summer due to large scale differences in temperature and associated biological growth and dormancy in a forested ecosystem. By observing seasonal stream chemical patterns when mean monthly streamflow is held relatively constant, insights into the relative importance of seasonal differences in temperature and biological activity, are gained. In this evaluation simplifying assumptions include (1) similar stream discharge values reflect similar flow paths and associated vertical source area and transit times within a watershed and (2) any differences in hydrologic storage minimally effected C–Q dynamics. A more detailed discussion of select assumptions is provided within the scope and limitations section. Here we discuss the relative importance of seasonal streamflow variability and seasonal distinctions in biogeochemical activity in determining characteristic seasonal water chemistry patterns based on stream observations. We further discuss the likely biogeochemical mechanisms contributing to those distinctions. We encourage the evaluation of similar atypical stream chemistry and discharge data sets, especially in watersheds where detailed internal catchment data are available, to document consistency or differences in patterns and assess the suggested driving mechanisms.

### The role of seasonal differences in biogeochemical processes and hydrological flow in stream chemistry patterns

The characteristic seasonal patterns for individual stream analytes used to assess the status of stream acidification are controlled by the roughly equivalent and additive impact of seasonal variability in hydrological flow and seasonal differences in biogeochemical activity. The most acidic conditions, defined by minimum ANC, occur during the characteristic winter with high-flow period due to the combined impact of lower base cations and elevated sulfate. Our results indicate that seasonal variations in biogeochemical processes play a significant role in controlling the typical intra-annual variability in stream chemistry, in addition to changes in hydrology and associated source area.

Despite the evolved understanding of the complex interactions driving bedrock derived stream solute concentrations, consideration of seasonal variability in weathering processes associated with oscillations in biological growth and dormancy in forested watersheds are not common. The basic conceptual framework underlying stream geogenic solute dynamics in stream water is one of variability in vertical source area characteristics (Fig. 1). Lower flows are derived from groundwater sources with elevated concentration due to interactions with actively weathering bedrock and longer transit and contact times (Jin et al. 2010; McIntosh et al. 2017; Stewart et al. 2021). Models have been used to investigate the role of transit time distribution (Torres and Baronas, 2020) and vertical connectivity (Xiao et al. 2021), as well as complex interactions of changes in subsurface hydraulic conductivity, weathering reaction kinetics and flow distinctions (Ameli et al. 2017) on bedrock derived solute patterns. Despite an absence of direct consideration of seasonal controls on ANC and base cation patterns, elevated concentrations in summer for all flow conditions are not unexpected. Abundant literature documents the importance of temperature, as well as precipitation and moisture, on weathering rates (Bluth and Kump 1994; White and Blum 1995; White et al. 1999; Kump et al. 2000; Millot et al. 2003). Plants and associated soil microbiota have also long been known to affect mineral weathering by lowering soil pH (Belt 1874) with the role of mycorrhizal fungi more recently acknowledged (Taylor et al. 2009). While biologically facilitated weathering is known to be highest during the growing season (Brantley et al. 2011), the importance of seasonal differences in biogeochemical processes, representing up to 78% of the increase from winter with high flow to summer with low flow in bedrock derived solutes, was surprising. Perhaps equally unexpected was the finding of consistent elevated concentrations of sulfate in winter (up to 40% higher than summer), regardless of streamflow. Consistent intra-annual sulfate patterns have not typically been linked to seasonal biogeochemical processes, unless driven by a hydrologic pattern (Mayer et al. 2010). However, changes in stream sulfate concentrations resulting from biological disturbance and unrelated to variations in hydrology or season have been documented (Nodvin et al. 1988; Webb et al. 1995; Eshelman et al. 1998).

The role of streamflow in determining analyte concentration conforms to expected patterns. Greater weathering rates resulting from greater surface area, longer contact time etc. as previously described at depth below the regolith provide the dominant source of base cations, silica, and ANC, therefore diluting with higher flow (Godsey et al. 2009; Ibarra et al. 2017; Moatar et al. 2017; Winnick et al. 2017; Zhi et al. 2019; Stewart et al. 2021). Conversely, sulfur content derived from atmospheric deposition is known to be elevated, or more available for transport due to adsorption reversibility, in surficial soils (Shanley 1992), therefore concentrating with higher flow. The atypical sulfate dynamics with flow at Paine Run (static in summer, dilution in winter), are likely related to a depletion of sulfate in the upper soils due to a more advanced state of recovery from acid deposition (Riscassi et al. 2019). However, this pattern is not evident in the quarterly data, which is more similar to mafic sites.

Although typically playing a minor role in acidification of streams in this region, nitrate and chloride are becoming more prominent anions as sulfate concentrations decline in response to emissions and associated deposition reductions (Eng and Scanlon 2021). The overall finding that seasonal biogeochemical processes have a prominent role, in addition to changes in flow, in controlling intra-annual nitrate variability was expected as nitrogen is an essential macro-nutrient. Despite the importance of both factors, the direction of their influence was inconsistent between bedrock types, and there was a large amount of variability in monthly concentrations, signified by the large concentration confidence intervals (Fig. 9e, f). Taken together, these observations indicate that the complicated interactions of nitrogen transformation and fluxes within the soil–plant-microbe system in forested watersheds are not easily simplified to monthly trends (Melillo et al. 1982; Lovett et al. 2000; Bohlen et al. 2001; Vitousek et al. 2002; Robertson and Groffman 2007; Mitchell 2011).

Chloride is the only analyte to demonstrate seasonal biogeochemical and hydrological flow effects that offset in a typical hydrologic year. At mafic sites, chloride dilutes with increases in stream flow, similar to bedrock-derived solutes, while concentrations are lower in summer for both bedrock types, similar to atmospherically derived solutes. Mafic bedrock has been associated with higher chloride concentrations in SHEN streams (Lynch and Dise 1985) and in forest soils in Sweden (Melkerud et al. 1992), therefore a significant bedrock source and greater weathering below the regolith is likely driving the negative relationship with flow observed at all mafic sites (Fig S3c). Weathering is not a candidate to explain lower summer concentrations, however, as other bedrock components such as SBC and silica are higher in summer. The lower growing season chloride concentrations indicate biogeochemical controls are present, but not associated with weathering, in line with recent research documenting extensive terrestrial cycling of chloride in forested watersheds (Lovett et al. 2005; Svensson et al. 2021). An alternative hypothesis to account for elevated chloride in winter is road salt additions. A main road runs through the upper reaches of both intensively studied watersheds and road salts are applied during icy conditions by the National Park Service. Chloride studies in the mid-Atlantic region have shown that watersheds with impervious cover < 10% do not demonstrate either sustained or short-term winter concentration increases (Moore et al. 2020). Impervious surface coverage for Piney River and Paine Run is < 0.1% and the road is in the upper reaches of the watershed ~ 1–2 km from the sample site location, therefore road salt is not a candidate to explain broad seasonal differences.

### Biogeochemical mechanisms driving seasonal differences

The consistency, significance, and occasional dominance of seasonal differences in biogeochemical activity in driving seasonal stream chemistry patterns highlights the need for insight into the likely processes. Below we evaluate potential mechanisms resulting in patterns of elevated concentrations of bedrock derived solutes, and depressed concentrations of sulfate and chloride, in the summer, compared to winter. Potential mechanisms leading to contrasting seasonal nitrate patterns between bedrock types are discussed in the following section.

Seasonal changes in stream concentrations of silica, base cations, and ANC (for the same streamflow) are likely a result of alterations in weathering rates of bedrock and minerals. Weathering rates increase in response to elevated temperatures and more acidic subsurface conditions (Schwartzman and Volk 1989; Berner 1992; White and Blum 1995). The dissolution rate of silicate minerals has experimentally been shown to increase with decreasing pH in the acidic range (< 4.5; typical of acid impacted regions) and mafic and basalt dissolution rates are expected to be greater than silicates (Drever 1994). Natural soil acidification occurs during the growing season due to root respiration and heterotrophic metabolism of soil organic matter which generates carbon dioxide (CO_2_), leading to the generation of carbonic acid (H_2_CO_3_) as well as through the production of organic acids (Brantley et al. 2011). Soil respiration has been shown to increase with net primary productivity (Andrews and Schlesinger 2001) and to vary seasonally with the highest respiration rates observed during the growing season at the Duke Forest (Raich and Schlesinger 1992) as well as at White Oak Run, a watershed adjacent to the Paine Run study site (Castelle and Galloway 1990). It is worth noting the seasonal differences in concentration for bedrock derived solutes were consistent for both lower and higher mean monthly flow conditions indicating a seasonal influence throughout the deeper and shallower subsurface. While interactions between biology and weathering are most intense and dynamic in the soil surface zone and therefore, we might expect a more dynamic higher flow signature, Brantley et al. (2011) emphasized that biological activity is also significant to weathering process in deeper zones of the regolith. Acids generated by biota can be transported beyond the site of origin to significant depths where they stimulate reactions (Oh and Richter 2005).

Both plant uptake and microbial immobilization as well as changes in soil acidity impacting anion adsorption in summer would result in reduced summer stream concentrations of sulfate and chloride. Sulfate uptake by forest vegetation, estimated between 2–3 kg ha^−1^ yr^−1^ (Johnson et al. 1982, 1984) corresponds in magnitude to differences in mass flux between seasons for mean flow (1.0 and 0.7 kg ha^−1^ yr^−1^at Piney River and Paine Run, respectively; Fig. S7a and b) indicating growing season uptake could be a reasonable mechanism to explain depressed summer concentrations. Natural soil acidification in summer, as previously described, could also be a reasonable mechanism driving lower stream concentrations by increasing soil sulfate retention. Nodvin et al. (1988) noted that the mechanisms of increased soil sulfate retention from soil acidification subsequent to deforestation in Hubbard Brook could also be relevant to seasonal trends in undisturbed forests. In contrast to sulfate, the processes and ecological roles of chloride cycling are relatively poorly understood (Svensson et al. 2021). However, chloride is known to be incorporated into the biotic environment (Chen et al. 2002; Johansson et al. 2003; Svensson et al. 2007; Clarke et al. 2009; Redon et al. 2011) which suggests depressed growing season concentrations could be linked to biological processes such as plant uptake and/or microbially driven chlorination of organic compounds (Lovett and Hubbell 1991; Bastviken et al. 2009).

In the absence of detailed subsurface catchment measurements, one way to evaluate if uptake or increased adsorption from natural acidification is likely responsible for observed seasonal dynamics of sulfate and chloride, would be to observe analyte behavior when the two mechanisms are competing. Fortunately, we have such observations subsequent to a biological disturbance in these watersheds. A regional multi-year spongy (previously ‘gypsy’) moth (*Lymantria dispar* L.), defoliation in the early 1990’s resulted in reduced vegetative uptake, and an increased rate of nitrification resulting in acidification of the soil (Eshelman et al. 1998). If plant uptake dynamics were a dominant control on sulfate and/or chloride, the expectation would be for an increase in stream concentrations during the disturbance. Stream concentrations would also be enhanced due to greater rates of organic matter mineralization as a result higher soil temperatures due to reduced vegetative shading. Sulfate concentrations notably decreased during the defoliation event (Webb et al. 1995), indicating that increased sulfate adsorption, associated with acidification of the soil, was most likely regulating stream concentrations. Unlike sulfate, stream chloride concentrations increased during the defoliation period, indicating that pH dependent adsorption was not a dominant mechanism. The chemical behavior observed during the ecological disturbance provide some indication of the likely mechanisms driving lower growing season concentrations; sulfate decreases in summer due to increases in soil acidity and associated adsorption dynamics and chloride decreases in summer due to plant uptake and microbial immobilization.

### Bedrock influence on seasonal stream chemical patterns

In general, bedrock-derived solutes are elevated during summer with low flow, sulfate is elevated during winter with high flow and chloride is similar in summer and winter, for both mafic and siliciclastic watersheds. Seasonal differences in hydrological flows and seasonal biogeochemical processes both contribute (between 25 and 78%) to typical seasonal variability at mafic and siliciclastic sites. This finding indicates changes to seasonal biogeochemical activity and/or hydrological flows will typically produce a similar direction of concentration change in either of the two bedrock types. Chloride is the only analyte for which similar seasonal behavior, a lack of pattern, is a result of different drivers, as previously described. As a result, significant changes in seasonal biogeochemical activity and/or hydrological flows in a future climate would be expected to impact seasonal chloride patterns and concentrations at mafic sites, but not siliciclastic, in this region.

Nitrate is the only analyte for which the winter with high flow to summer with low flow patterns are distinct between bedrock types, illustrated in both the high frequency (Fig. 9e, f) and quarterly (Fig. S6e, f) datasets; mafic sites have significantly depressed summer concentrations relative to winter, whereas siliciclastic sites illustrate more subtle seasonal variations with lower winter concentrations. Depressed concentrations in the summer with low-flow conditions and higher concentrations in the winter with high-flow conditions, have been observed and typically attributed to both growing season plant and microbial uptake (Mitchell et al. 1992; Wright et al. 2001; McHale et al. 2002; Dittman et al. 2007; Barnes et al. 2008; Sebestyen et al. 2008; Cai et al. 2011). Elevated summer or suppressed winter concentrations have also been observed, attributed to increased rates of N retention via terrestrial and in-stream immobilization in winter (Mulholland 2004; Roberts and Mulholland 2007), mineralization and nitrification outpacing uptake in summer (Swank and Vose 1997; Goodale et al. 2009), and complex riparian processes associated with microtopography (Duncan et al. 2015). While factors such as climate, soil type, and forest age may influence watershed nitrogen cycling and resultant seasonal stream nitrate patterns, characteristics associated with watershed bedrock appear to play an important role in these mid-Appalachian watersheds.

Two potential characteristics that could lead to distinct seasonal nitrate patterns associated with bedrock include hydraulic conductivity (Burns et al. 1998), and forest disturbance (Swank and Vose 1997). Hydraulic conductivity is higher at siliciclastic sites, therefore soil water nitrate from the dormant season could be more readily transported out of reach of vegetation in summer resulting in the observed slightly elevated nitrate in the growing season. While nitrate enriched groundwaters in northern US forests are rare (Sebestyen et al. 2019), Burns et al. (1998) noted that the fractured flow discharges, leading to high summer stream nitrate concentrations in the Catskills, have also been reported for regions underlain by sedimentary bedrock such as the Appalachian Mountains. Alternatively, in watersheds with elevated rates of tree disturbance or mortality, increased light penetration and warmer soil temperatures stimulating mineralization and nitrification in winter and new growth increasing summer demand, would produce the nitrate pattern of elevated winter and depressed summer concentrations, observed at mafic sites. Multiple exotic forest pests and pathogens currently impact watersheds within the Blue Ridge Mountain ecosystem (Anderson-Teixeira et al. 2021). If disturbance is preferentially occurring to vegetation in mafic watersheds, it could explain the pronounced seasonal pattern of depressed summer and elevated winter concentrations. While contrasts in seasonal nitrate patterns have not yet been assessed with respect to bedrock, parent material is known to be a dominant environmental factor influencing major vegetation patterns in SHEN (Young et al. 2006). While the specific mechanism is unknown, differences in bedrock as they result in variations in watershed characteristics, result in distinct seasonal nitrate patterns, in contrast to all other analytes evaluated.

## Scope and limitations

The finding that seasonal differences in biogeochemical processes and hydrological flow are similarly important in determining seasonal stream chemistry for a variety of analytes is likely broadly applicable to temperate forested mountain watersheds. However, there will necessarily be differences due to factors such as climate and soil properties. For example, watersheds in the northeastern U.S. which have a consistent winter snow cover which insulates soils and a more moderate summer climate, may have less seasonal variability in soil temperature, compared to the mid-Appalachian region. As a result, seasonal biogeochemical processes driven by differences in soil temperature may not be as distinct between winter and summer seasons, and therefore play a lesser role in seasonal patterns. Soil properties are also known to be distinct regionally, with sulfate adsorption being weaker in the glaciated northeastern region (Eng and Scanlon 2021) and as a result, seasonal changes in soil pH, may have different impacts on sulfate dynamics.

We evaluate relationships between mean monthly flow and mean flow-weighted analyte concentration on a seasonal timeframe without consideration of hydrological and biogeochemical interactions resulting from differences in storage. Climate-related variability in watershed storage has been shown to influence C–Q relationships in a forested watershed in the southeastern US, with the greatest impact observed for reactive solutes such as sulfate, and minor impacts on weathering products and chloride (Aulenbach 2020). Our results support the general finding that watershed biogeochemical processes are important in determining stream water sulfate concentrations and expand the drivers of those processes to include seasonal variability in temperature and biological activity. In this evaluation we assume similar mean monthly flows reflect similar subsurface hydrological flow paths and interactions and linkages with biogeochemical processes such as transit and reaction times regardless of season. While this assumption is more reasonable for low flow conditions, we acknowledge there are likely significant distinctions in hydrologic characteristics (e.g., flow path, contact time) associated with the same monthly high flow in summer and winter. In summer, high monthly streamflow results from frequent precipitation events in conjunction with evapotranspiration, which will produce large fluctuations in the water level, while the same monthly flow during winter would be the result of moderate precipitation and low evapotranspiration and produce a relatively stable water level. Watershed wetting and drying cycles regulate the balance of moisture and oxygen in the soil. Variations on the timing of those cycles, have been shown to influence biogeochemical processes, such as soil respiration rates and soil CO_2_ diffusivity (Welsch and Hornberger 2004) and stream chemical concentrations of sulfate (Mayer et al. 2010) and nitrate (Robertson and Groffman 2007; Duncan et al. 2017). Despite this limitation, we have observed that mean monthly flow-weighted analyte concentrations can be significantly different between summer and winter, given the same mean monthly flow conditions, typically observed in the winter season.

## Implications

Findings from this work have implications for seasonal biogeochemical model parameterization and some C–Q interpretations. Integrated hydrologic and biogeochemical models such as PnET-BGC (Photosynthesis-EvapoTranspiration and BioGeoChemistry), incorporate weathering and soil sulfate adsorption, as well as vegetation element uptake processes and were designed to assess land disturbance, such as a changing climate, superimposed on acidification recovery. While PnET-BGC applied to southeastern watersheds has demonstrated stream ANC is particularly sensitive to the partial pressure of carbon dioxide (*p*CO_2_;Gbondo-Tugbaw et al. 2001) as well as Ca and Mg weathering rates (Fakhraei et al. 2017), those processes have often been parameterized as static (Zhou et al. 2015). Our results suggest that seasonally varying *p*CO_2_ should be included in watershed models to simulate seasonal changes in weathering and sulfate adsorption rates and resultant stream acidity. Stream flow and chemistry data collected during atypical hydrologic years, such as those presented in this study, can also be used to verify accurate model parameterization. Appropriate attribution of seasonal stream chemical drivers will provide confidence in predictions of how changes in those same drivers in a future climate (e.g. lower winter streamflow or shift in timing of spring phenology) will impact stream chemical conditions.

The finding that mean monthly C–Q intercepts and slopes are distinct rather than matching between seasons, when evaluated over the same mean monthly discharge range, advances the interpretation of C–Q evaluations conducted with regular sampling throughout the year in environments where streamflow and season are synchronous. A positive C–Q is typically characterized as a ‘transport limited’ or ‘flow mobilized’ solute, a negative C–Q as ‘source limited’ or ‘flow diluted’ and no significant slope as ‘chemostatic behavior’ indicating ample, consistent sources (Godsey et al. 2009; Musolff et al. 2015; Moatar et al. 2017). This interpretation does not directly consider that flow may be associated with season. Our findings indicate the magnitude of the positive and negative slope can be influenced by seasonal differences in biogeochemical activity, in addition to stream flow generation processes. For example, the significant negative silica C–Q in a typical hydrologic year is due to both lower winter concentrations superimposed on depressed high-flow concentrations. Furthermore, chemostatic behavior may be a result of offsetting seasonal and hydrological flow dynamics such as observed for chloride at Piney River and other mafic sites. Future assessments of concentration changes with stream flow should acknowledge that seasonal distinctions in biogeochemical processes and seasonal hydrological flow may act in concert to amplify and/or dampen those dynamics.

Observations of stream chemistry during atypical seasonal patterns in hydrology may be useful to test current, or inform future, integrated theories of biogeochemical reaction kinetics and hydrological controls at the catchment scale. As climate change is playing out in real time, the ability to elucidate controlling processes on stream geochemistry to prepare for the future depends on insights gained from data collection platforms such as those used in this study. Long-term monitoring has guided resource management decisions and environmental policy to success in the past (Lovett et al. 2007; Sullivan et al. 2018) and should be maintained to address the current and unknown threats of the future.

## Supplementary Information

Below is the link to the electronic supplementary material.

Supplementary file1 (DOCX 3997 KB)

## Data Availability

All water chemistry data are available through the Water Quality Portal, a cooperative service sponsored by the U.S. Geological Survey, U.S. Environmental Protection Agency, and the National Water Quality Monitoring Council (https://www.waterqualitydata.us/) (Project ID: SHEN_UVA_PRIMARY). Time series data, including hourly stream discharge, are available through the National Park Service Aquarius Web Portal https://irma.nps.gov/aqwebportal/. Sites and associated data are accessed using identifier prefix SHEN_SWAS_SITEID (where SITEIDs are PAIN and PINE).
