# Supplementary material for: Factors influencing seasonal chemistry patterns in Virginia mountain streams
Source: Biogeochemistry. 2024 Aug 6;167(10):1175–201. doi: 10.1007/s10533-024-01163-x (PMC11489209; doi:10.1007/s10533-024-01163-x)
Supplement: Supplementary file 1 — Supplementary file1 (DOCX 3997 KB) [file 10533_2024_1163_MOESM1_ESM.docx]

Supplementary Information for

Factors influencing seasonal chemistry patterns in Virginia mountain streams

Riscassi, Ami L.^1*^, Scanlon, Todd M.^1^, and Galloway, James N.^1^

^1^ University of Virginia, Environmental Sciences Department, Charlottesville, Virginia, US

^*^ Corresponding Author: [alr8m@virginia.edu](mailto:alr8m@virginia.edu)

Supplemental Text (2)

Supplemental Figures (8)

Supplemental Tables (1)

References

Supplementary Information includes descriptions and presentations of supporting data.

A description of atmospheric deposition fluxes (supplemental text 1) and the presentation of annual watershed input and output fluxes at the two study sites for each of the three water years (Fig. S3) are provided to illustrate the magnitude of inter year flux differences. A description of quarterly sites and data synthesis (supplemental text 2), quarterly sample site locations (Fig. S1), and regional precipitation maps (Fig. S2) are provided to describe and support the quarterly stream concentration summary for 2016-2018 water years (Fig. S6). The supplementary table (Table S1) includes statistics for the monthly C-Q relationships determined for winter and summer seasons presented in the manuscript. Instantaneous concentration discharge (C-Q) data and best fit lines for the summer and winter season for the 2016-2018 water years (Figs. S4 and S5) are provided for comparison to monthly data presented in the manuscript. Monthly streamflow vs analyte stream flux (Figs. S7 and S8) for the summer and winter seasons are presented for each analyte to illustrate the magnitude of monthly flux differences between seasons.

**Supplemental Text**

**Text S1) Monthly atmospheric deposition fluxes - Methods**

Monthly wet deposition for the 2016, 2017, and 2018 water years at Piney River and Paine Run was calculated from monthly precipitation and analyte concentrations obtained from the National Atmospheric Deposition Program/National Trends Network (NADP/NTN) Big Meadows site (VA28) located in central Shenandoah National Park (SHEN; Fig. S1). Monthly wet deposition for each watershed was estimated by multiplying the wet deposition flux at VA28 by the ratio of precipitation for the respective watershed to that of VA28. Monthly precipitation data was obtained for each site from the Parameter-elevation Regression on Independent Slopes Model which has a ~4 km resolution (PRISM, http://prism.oregonstate.edu, accessed 2020-Feb) using the ‘interpolation option’ with the centroid of the watershed as the ‘point’ of the estimation. Monthly total deposition fluxes (wet + dry) were calculated by multiplying the wet deposition flux by the annual ratio of total/wet deposition for each analyte at each watershed, available from the NADP total deposition (TDEP) ESRI Arcgrids at calendar year resolution (TDEP v. 2018.2 <http://nadp.slh.wisc.edu/committees/tdep/> ). Of note, because total N wet deposition was not available at monthly resolution, monthly total N flux was determined by multiplying monthly N as nitrate in wet deposition by the ratio of total N to N as nitrate in wet deposition from the annual TDEP Arcgrids. Methods for estimation of TDEP data are detailed in Schwede and Lear (2014). Monthly total deposition fluxes were aggregated to water year and illustrated in Figure S3.

**Text S2) Quarterly concentrations – Methods**

Analyte concentrations for samples collected at sites sampled quarterly as part of the SWAS-VTSSS program were compared for the last week in January (i.e., winter) and the last week in July (i.e., summer) of the 2016/2017 and 2018 water years. Only sites in which samples were collected in each year for each season were included in the analysis. Sites were grouped based on dominant bedrock, which included 5 sites underlain with mafic bedrock (including Piney River) and 35 underlain by siliciclastic bedrock (including Paine Run), illustrated in Figure S1.

Stream discharge was not measured at sites sampled only at quarterly resolution; therefore, concentrations are not flow-weighted. The hydrologic conditions documented at the two intensive sites were evaluated throughout the quarterly sampling region by observing deviation maps from the long-term average precipitation for the four months preceding quarterly sampling for the 2016-2018 water years. The precipitation deviation trends are consistent for the larger study site region, except for the southwest corner of Virginia in 2018 (Fig. S2). To confirm results were not biased by sites experiencing different seasonal precipitation trends, statistical differences were calculated for all siliciclastic sites (there are no mafic sites in that region) and those within SHEN. Though statistical significance was not always consistent, patterns were found to be similar. Data for the entire siliciclastic data set are presented in Figure S6.

Each bedrock class has four data groupings. For summer, there was a typical ‘low flow’ group, represented by the 2016/2017 water years and an anomalous ‘high flow’ group, represented by the 2018 water year. Similarly, for winter there was an anomalous low flow group, represented by the 2018 water year, and a typical high flow group represented by 2016/2017 water years. For each analyte and each bedrock type the resultant four sets of data were evaluated to determine if they met the assumptions of homoscedasticity (using Cochran’s test for equality of variance) and normality (using Jarque-Bera test for goodness-of-fit to a normal distribution). If data met both assumptions, an ANOVA was used, if not, the Kruskal-Wallis test was used to determine the level of differences between data sets. If differences were significant, a post-hoc test (tukey-kramer) was used to determine which groups were significantly different from each other.

**Supplemental Figures**


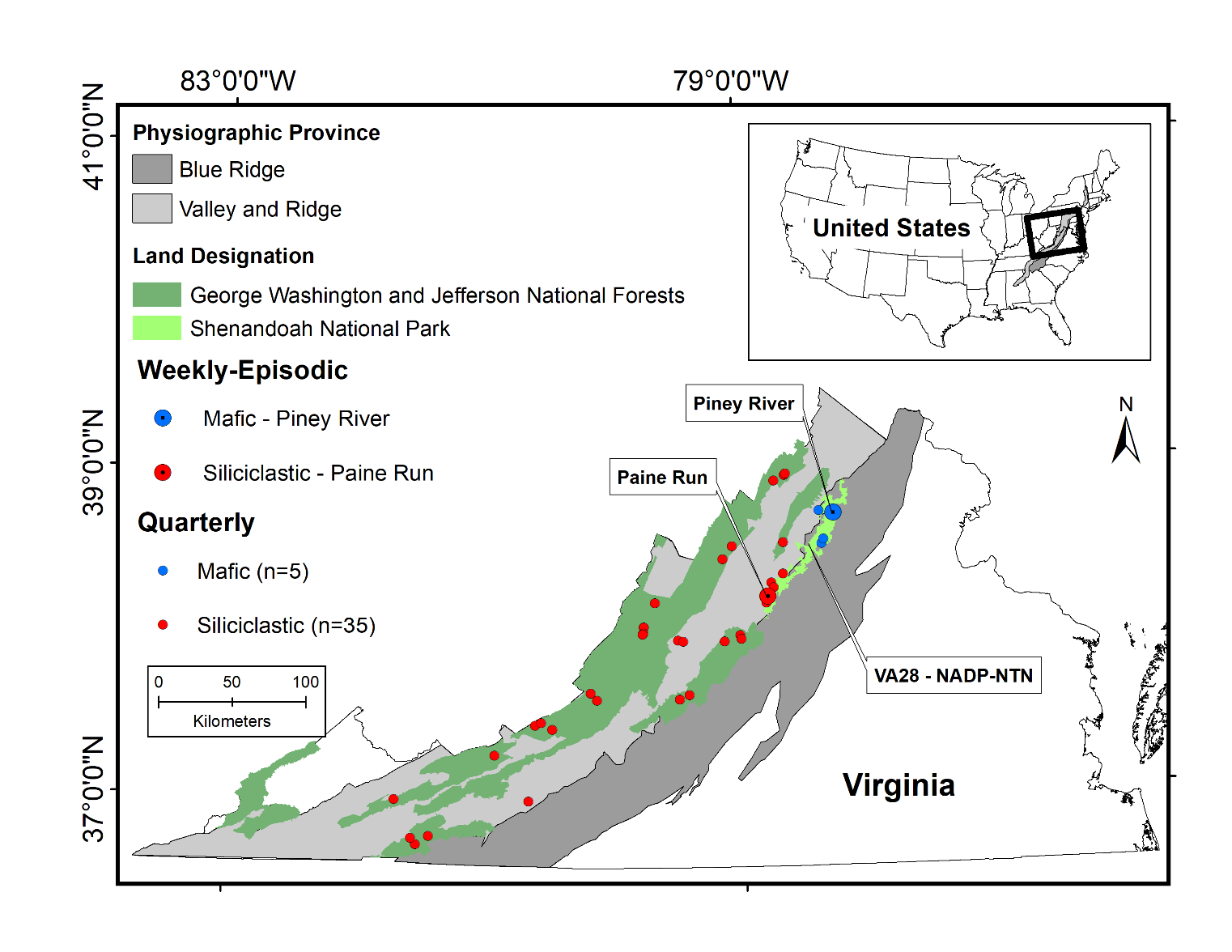


**Figure S1.** Map of study sites, distinguished by sampling frequency, land designation, and physiographic province. The National Atmospheric Deposition Program/National Trends Network (NADP/NTN) station, located in central Shenandoah National Park (VA28), is also indicated.


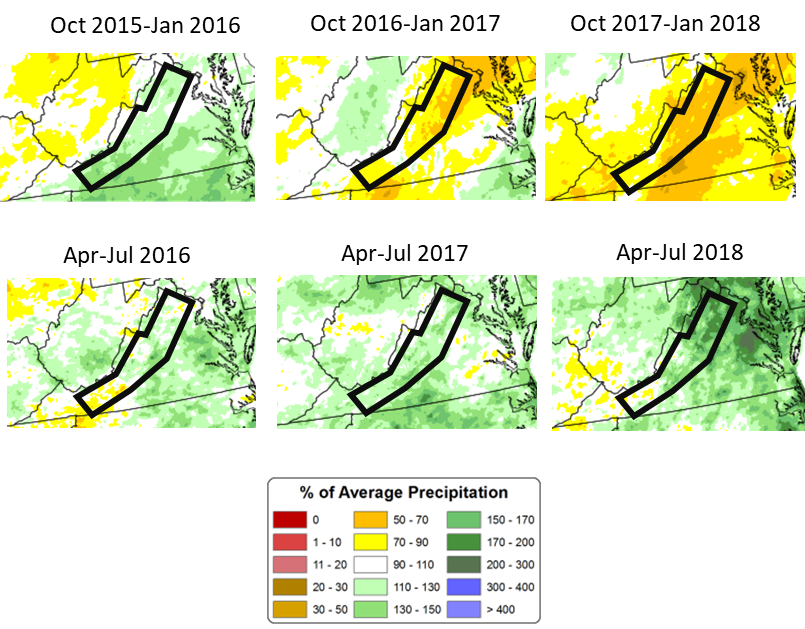


**Figure S2.** Map of total precipitation deviation from the 30-yr average (1991-2020) for the four months preceding winter (upper row) and summer (lower row) quarterly sampling for each of the three water years evaluated. The black outline represents the region where quarterly sampling sites are located. All images are from the PRISM Climate Group, Oregon State University (obtained April 26, 2022).

**
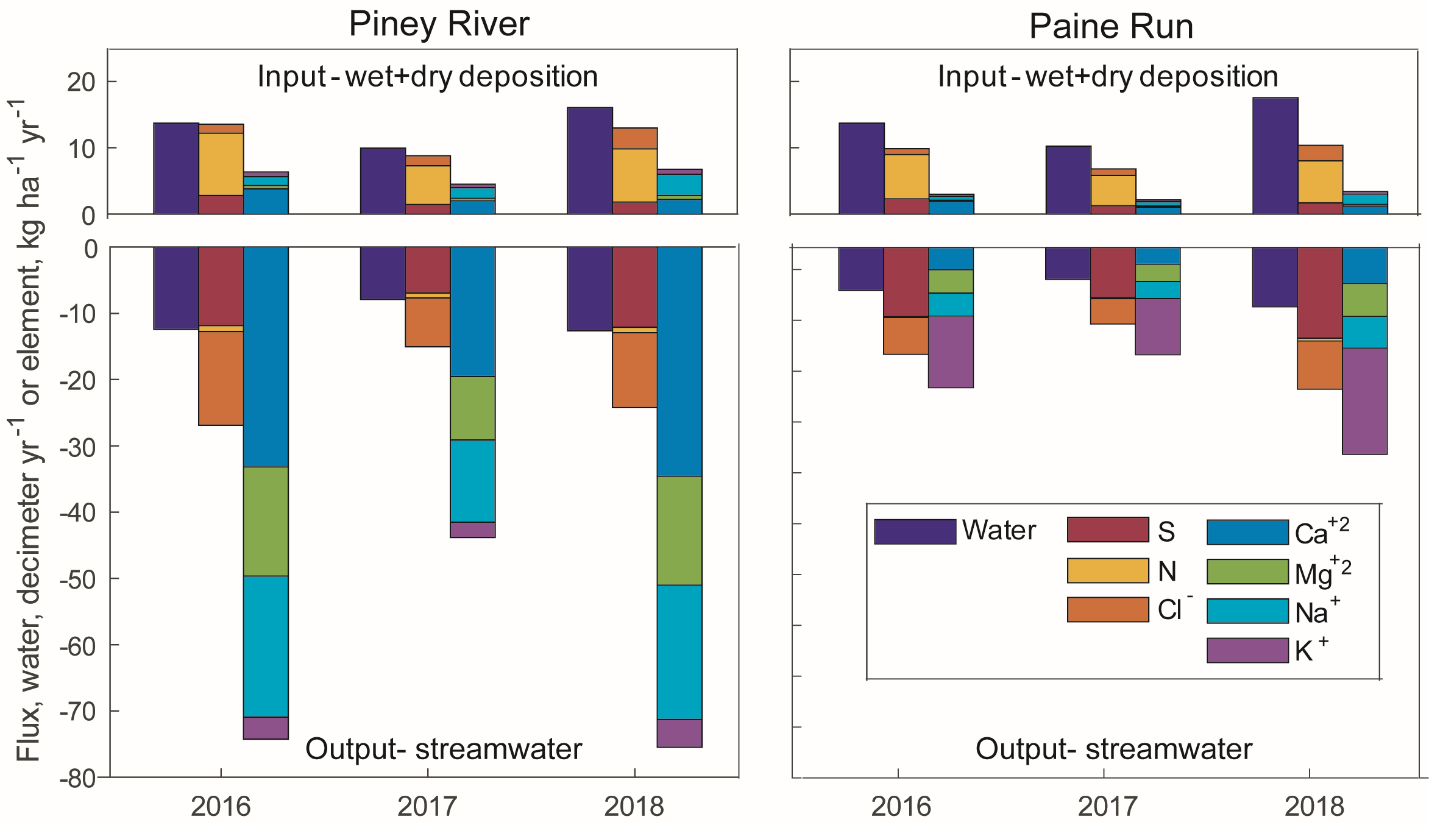
Figure S3.** Watershed input and stream output fluxes for water, sulfur (S), nitrogen (N), chloride (Cl^-^), calcium (Ca^+2^), magnesium (Mg^+2^), sodium (Na^+^) and potassium (K^+^) at Piney River (left panel) and Paine Run (right panel) for the 2016, 2017, and 2018 water years.

**
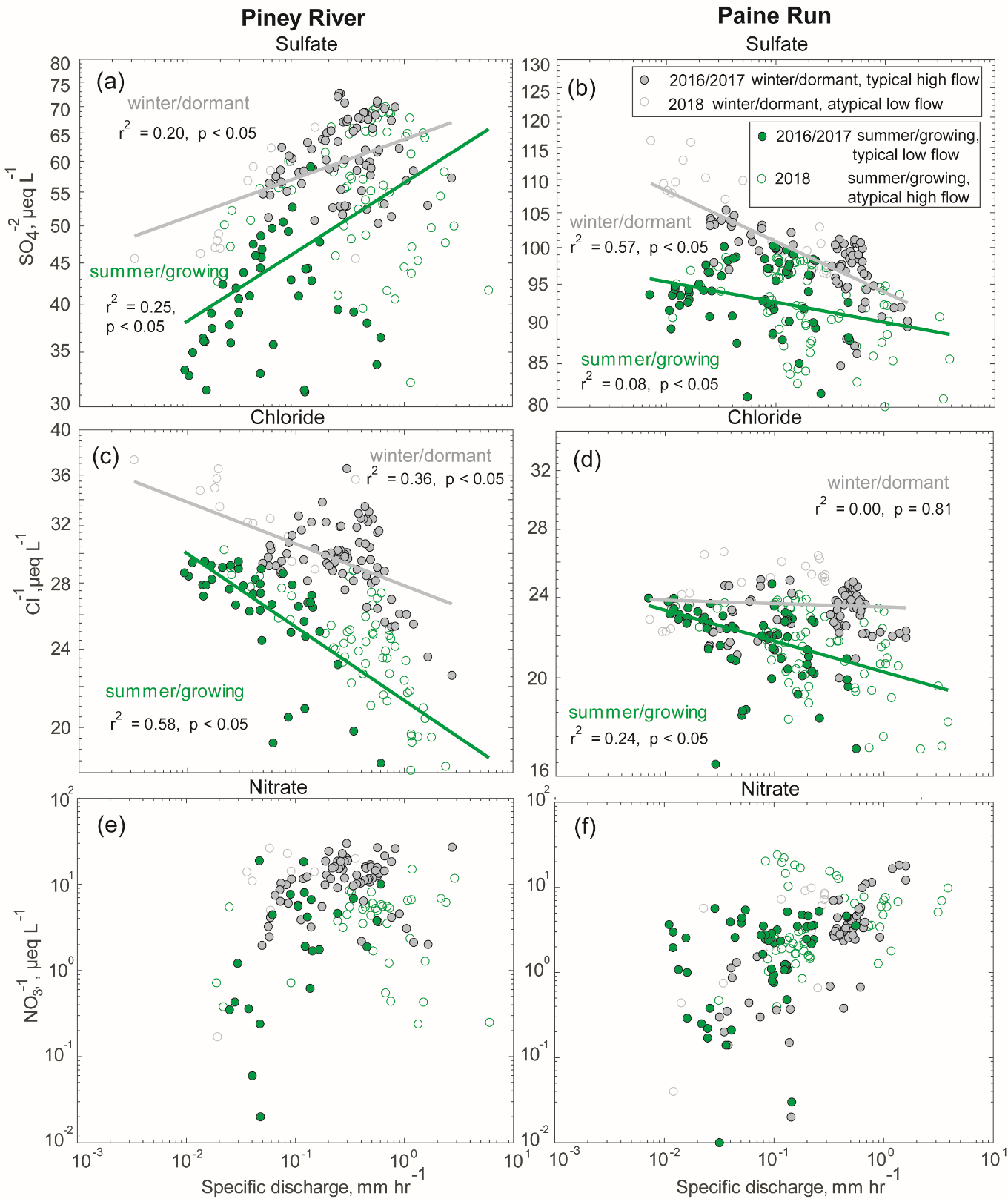
**

**Figure S4.** Instantaneous concentration versus specific discharge for the summer, growing season and winter, dormant season for (a, b) sulfate (SO_4_^-2^), (c, d) chloride (Cl^-^), and (e, f) nitrate (NO_3_^-^) at Piney River (left column) and Paine Run (right column). The best fit lines for the respective seasons, inclusive of both the typical (closed circles) and atypical (open circles) water years, are presented along with the associated r^2^ and *p* values. No lines are provided for nitrate due to a significant amount of data being below detection limit, as described in the manuscript.

**
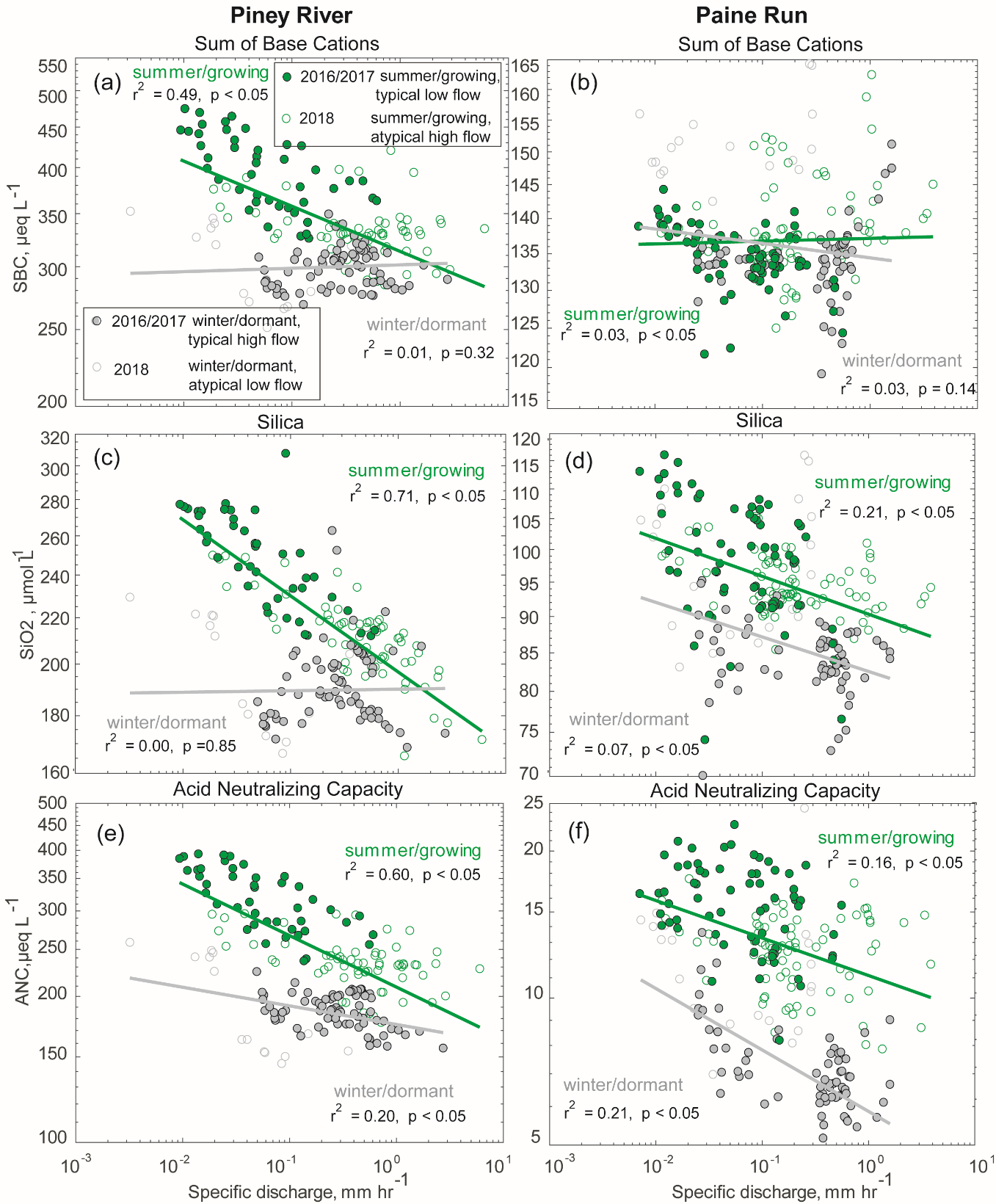
**

**Figure S5.** Instantaneous concentration versus specific discharge for the summer, growing season and winter, dormant season for the (a, b) sum of base cations (SBC), (c, d) silica (SiO_2_), and (e, f) acid neutralizing capacity (ANC) at Piney River (left column) and Paine Run (right column). The best fit lines for the respective seasons, inclusive of both the typical (closed circles) and atypical (open circles) water years, are presented along with the associated r^2^ and *p* values.

**
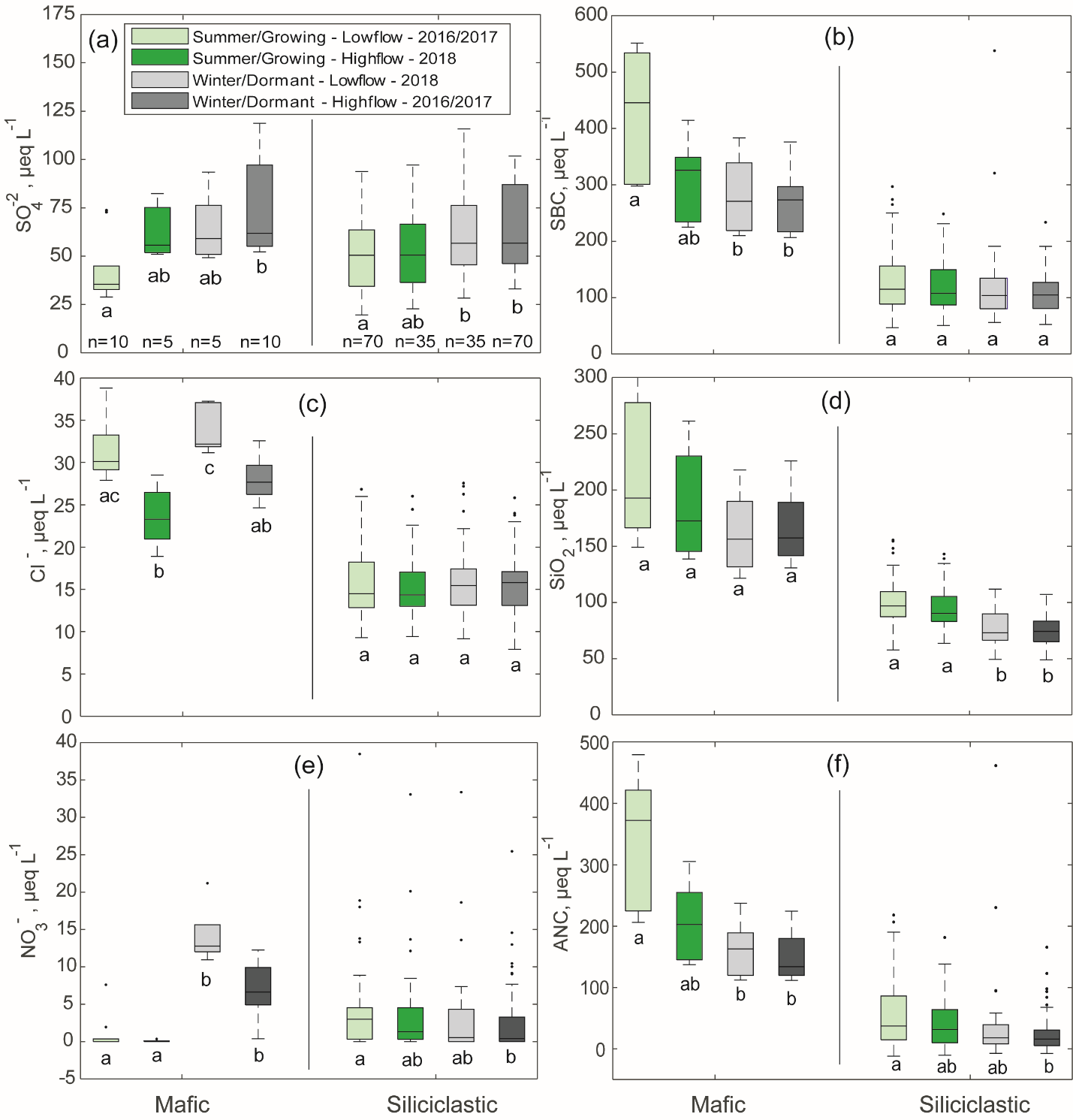
Figure S6.** Box whisker plots (center line = median, box limits = 25 and 75^th^ percentiles, outer lines = minimum and maximum, dots are outliers) of quarterly data at mafic (5 sites) and siliciclastic (35 sites) sites sampled quarterly for (a) sulfate (SO_4_^-2^), (b) sum of base cations (SBC), (c) chloride (Cl^-^), (d) silica (SiO_2_), (e) nitrate (NO_3_^-^), and (f) acid neutralizing capacity (ANC). Within each bedrock class, data are grouped by season and water year representing either typical or atypical hydrologic conditions as noted in the legend. Unique letters indicate significant differences between data sets for a bedrock class.

**
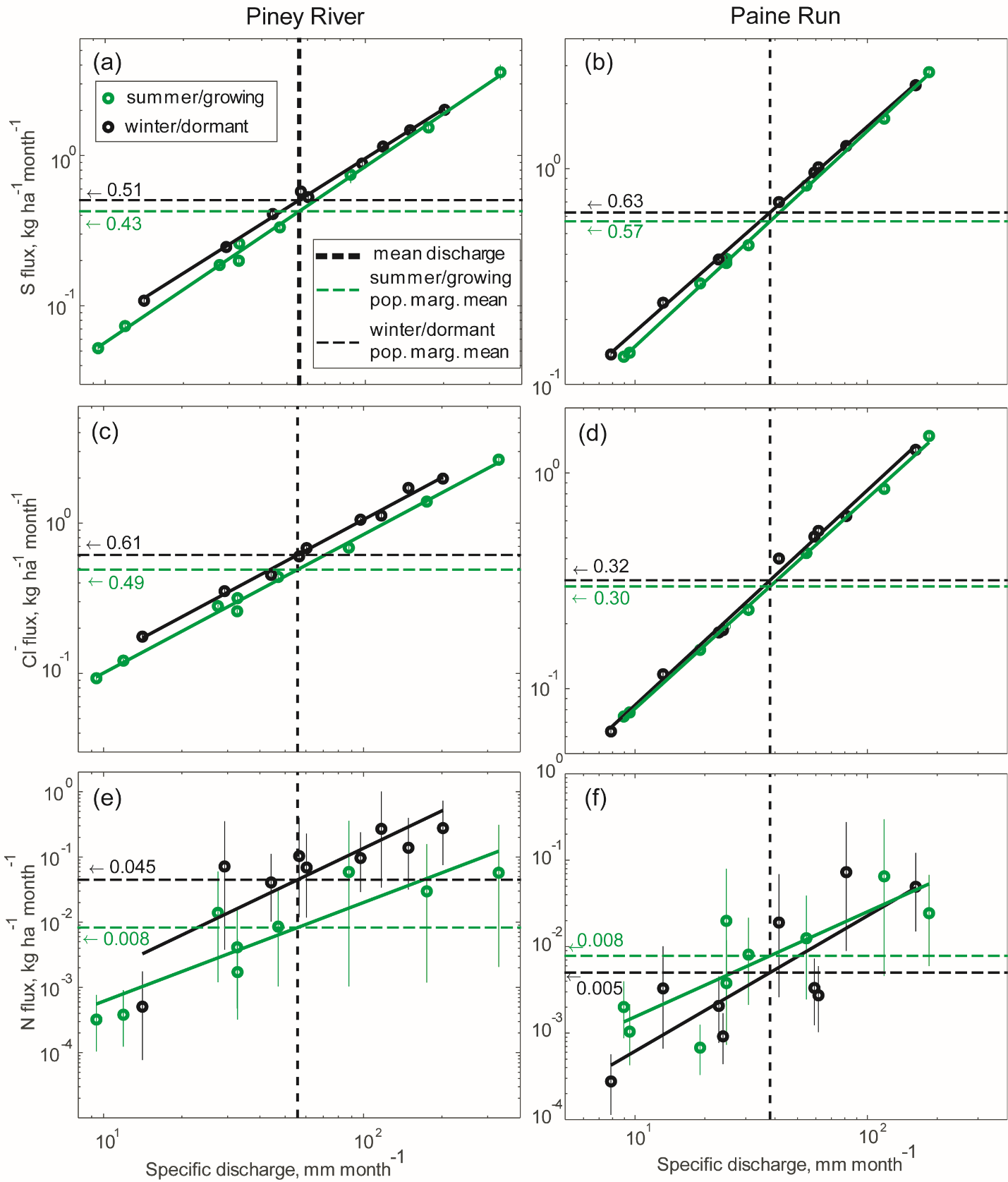
**

**Figure S7.** Monthly discharge for the summer, growing season and winter, dormant seasons verses the monthly analyte flux for (a, b) sulfur (S), (c, d) chloride (Cl^-^) and (e, f) nitrogen (N) flux at Piney River (left column) and Paine Run (right column). The flux associated with the population marginal mean for each season is indicated with a dashed line with the numerical value provided as text, both colored accordingly to the respective season.

**
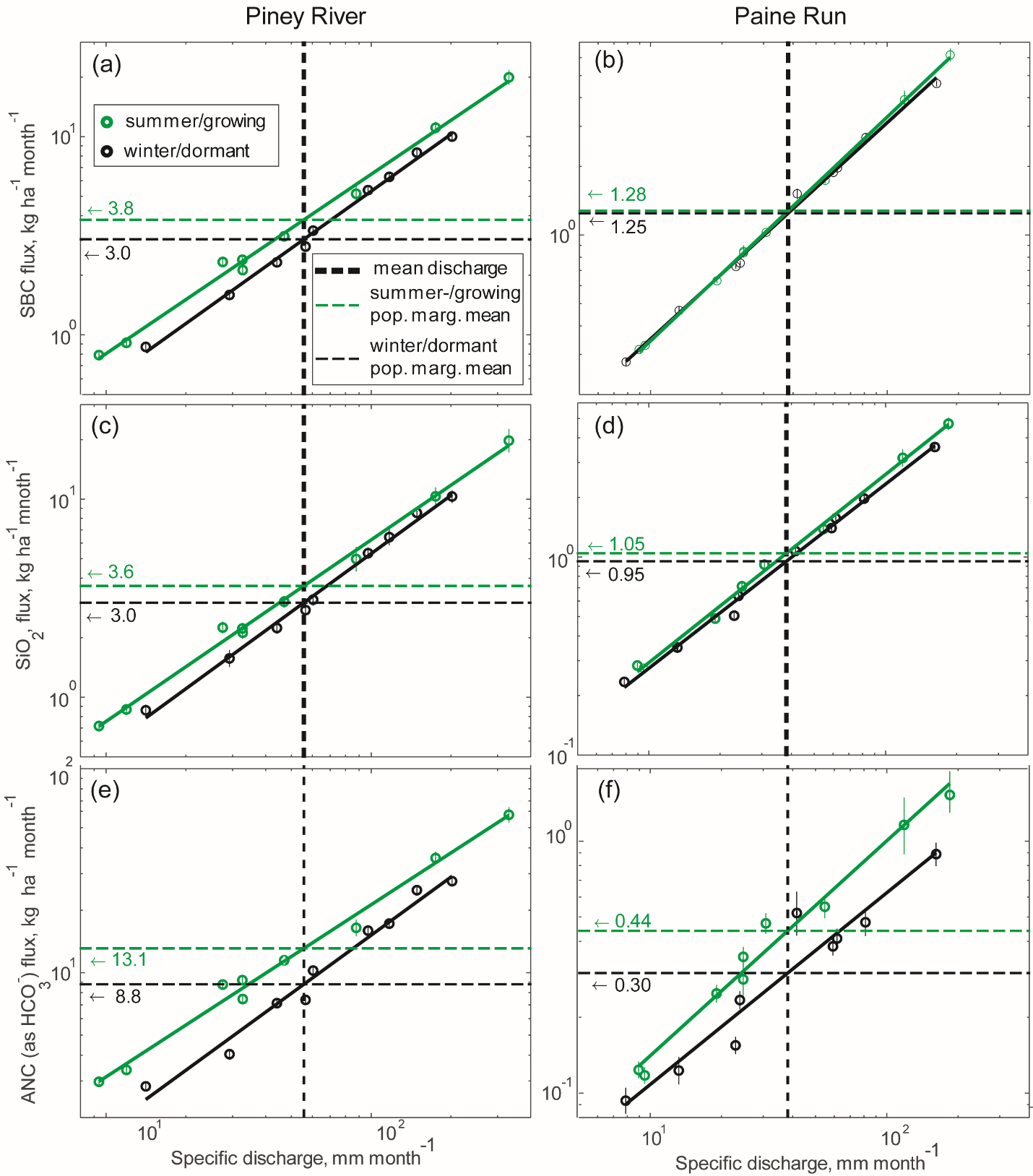
**

**Figure S8.** Monthly discharge for the summer, growing season and winter, dormant season verses the monthly analyte flux for the (a, b) sum of base cations (SBC), (c, d) silica (SiO_2_) and (e, f) ANC (calculated as bicarbonate, HCO_3_^-^) flux at Piney River (left column) and Paine Run (right column). The flux associated with the population marginal mean for each season is indicated with a dashed line with the numerical value provided as text, both colored accordingly to the respective season.

**Supplemental Tables**

**Table S1.** Slope and intercept of monthly concentration vs discharge (C-Q) best fit lines for winter and summer for individual analytes at the two study watersheds. Equations were calculated with discharge units of mm month^-1^ and concentration units of µeq L^-1^ with the exception of silica (µmol L^-1^). The concentration at mean flow (i.e., population marginal mean) and associated significance of differences between seasons for each statistic is also presented. Insignificant slopes (*p* values are presented in figures) are presented in grey. SBC, Sum of base cations, ANC, Acid Neutralizing Capacity.

**References**

Schwede DB, Lear GG (2014) A novel hybrid approach for estimating total deposition in the United States. Atmos Environ 92:207–220. <https://doi.org/10.1016/j.atmosenv.2014.04.008>
